# Reversible aggregation-redispersion of Cu sites in Cu/CeO_2_ catalysts with unlocked hydrogenation activity

**DOI:** 10.1126/sciadv.aed2774

**Published:** 2026-03-27

**Authors:** Yu Zhang, Ningqiang Zhang, Yiwei Liu, Haofan Lei, Tao Zhou, Wenlong Wu, Wei-Wei Wang, Han Yan, Chao Ma, Ken-ichi Shimizu, Chun-Jiang Jia, Jie Zeng

**Affiliations:** ^1^ Hefei National Research Center for Physical Sciences at the Microscale, Key Laboratory of Strongly-Coupled Quantum Matter Physics of Chinese Academy of Sciences, Key Laboratory of Surface and Interface Chemistry and Energy Catalysis of Anhui Higher Education Institutes, Department of Chemical Physics, University of Science and Technology of China, Hefei, Anhui 230026, P. R. China.; ^2^ SEP Key Laboratory of Eco-industry, School of Resources and Civil Engineering, Northeastern University, Shenyang 110819, P. R. China.; ^3^ Institute for Catalysis, Hokkaido University, Sapporo 001-0021, Japan.; ^4^ School of Chemistry, Dalian University of Technology, Dalian, Liaoning 116024, P. R. China.; ^5^ School of Chemistry and Chemical Engineering, Anhui University of Technology, Ma’anshan, Anhui 243002, P. R. China.; ^6^ Key Laboratory for Colloid and Interface Chemistry, Key Laboratory of Special Aggregated Materials, School of Chemistry and Chemical Engineering, Shandong University, Jinan, Shandong 250100, P. R. China.; ^7^ College of Materials Science and Engineering, Hunan University, Changsha, Hunan 410082, P. R. China.

## Abstract

For oxide-supported metal catalysts, metal-support interaction (MSI) facilitates metal dispersion at the expense of the metallic character, resulting in a trade-off between active site utilization and intrinsic activity. Here, we used a thermal aging strategy to modulate the MSI in Cu/CeO_2_ catalysts, facilitating the formation of metallic Cu sites upon H_2_ reduction while maintaining metal dispersion. Systematic experiments confirmed that thermal aging at 800°C lowered the reduction temperature and increased the reduction degree of Cu sites. Microscopy evidenced few-atom-layered Cu nanoclusters before and after H_2_ reduction, whereas in situ spectroscopy revealed metallic Cu nanoparticles under H_2_ atmosphere. This discrepancy indicated a reversible structural evolution from aggregation to redispersion in thermally aged Cu/CeO_2_. The catalytic activity for acetylene semihydrogenation was unlocked on metallic Cu sites, compared to nearly inactive Cu sites in conventional Cu/CeO_2_ counterparts. Our work developed an effective strategy for rational modulation of MSI, offering the feasibility to tailor-make active sites for specific reactions.

## Teaser

Thermal aging unlocks the hydrogenation capability of inactive Cu species on CeO_2_ via a dynamic structural change.

## INTRODUCTION

Supported metal catalysts play a pivotal role in catalysis, with wide applications across diverse heterogeneous catalytic processes, such as CO_2_ hydrogenation (*1*–*3*), water-gas shift (*4*–*6*), and methane dry reforming (*7*–*9*). Although the active sites are typically associated with the supported metal, the properties of these sites are profoundly influenced by support via the metal-support interaction (MSI) (*10*–*13*). MSI dictates the electronic structure (*14*), geometric configuration (*15*), and chemical composition of the supported metal (*16*). Meanwhile, MSI governs numerous dynamic phenomena, including spontaneous redispersion (*17*–*19*), spillover (*20*, *21*), and metal encapsulation (*22*), complicating the catalyst system. Therefore, understanding and elucidating the role of MSI in catalysis is challenging, and modulating it is even more so. Furthermore, deciphering these MSI-induced dynamic processes, particularly under in situ conditions, proves essential for bridging fundamental comprehension with the rational design of active sites.

Among the catalyst support materials, CeO_2_ stands out due to its distinctive properties, such as the oxygen vacancy formation (*23*), facet effect (*24*), and solid solution formation (*25*), leading to the unique interaction between CeO_2_ and supported metals (*26*–*28*). Such MSI facilitates the formation of metal─O─Ce bonding (*29*), endowing CeO_2_ with an excellent capability to disperse metals (*30*, *31*). Specifically, the strong Cu-CeO_2_ interaction (represented as Cu─O*_x_*─Ce) enables high loading of well-dispersed Cu species, contributing to the application of Cu/CeO_2_ catalysts in specific reactions (*32*–*34*). However, the resultant high oxidation state of Cu sites impairs broader catalytic application, calling for strategic modulation of MSI to balance the dispersion and metallic character of supported Cu sites. Moreover, investigating the accompanying dynamic structural evolution will deepen the fundamental comprehension of MSI, paving the way for rational catalyst design.

Here, we used a thermal aging strategy to modulate the MSI in Cu/CeO_2_ catalysts, facilitating the formation of metallic Cu sites during H_2_ reduction while maintaining high metal dispersion. A series of experiments demonstrated that thermal aging at 800°C provided an optimal condition, which lowered the reduction temperature of Cu species while preserving their high dispersion. Microscopy analysis showed that, in thermally aged Cu/CeO_2_, Cu remained in the form of highly dispersed, few-atom-layered clusters before and after H_2_ reduction. On the other hand, in situ spectroscopy revealed metallic Cu nanoparticles with a high coordination number (CN) under H_2_ atmosphere. This conflict indicated a reversible structural evolution, with oxidized Cu clusters aggregated into metallic particles during H_2_ reduction and redispersed into partially oxidized clusters upon air exposure. Thermal aging unlocked the hydrogenation activity of Cu, as validated by higher H_2_-D_2_ exchange rates and enhanced acetylene semihydrogenation performance. At 200°C, while conventional Cu/CeO_2_ exhibited low C_2_H_2_ conversion (31.1%), similar to that of bare CeO_2_ support (35.8%), thermally aged Cu/CeO_2_ achieved both high conversion (98.8%) and selectivity (95.5%). Our work developed an effective strategy to rationally modulate MSI, potentially expanding the application boundaries for CeO_2_-supported metal catalysts.

## RESULTS

### Thermal aging modulates the MSI in Cu/CeO_2_

**Fig. 1.**
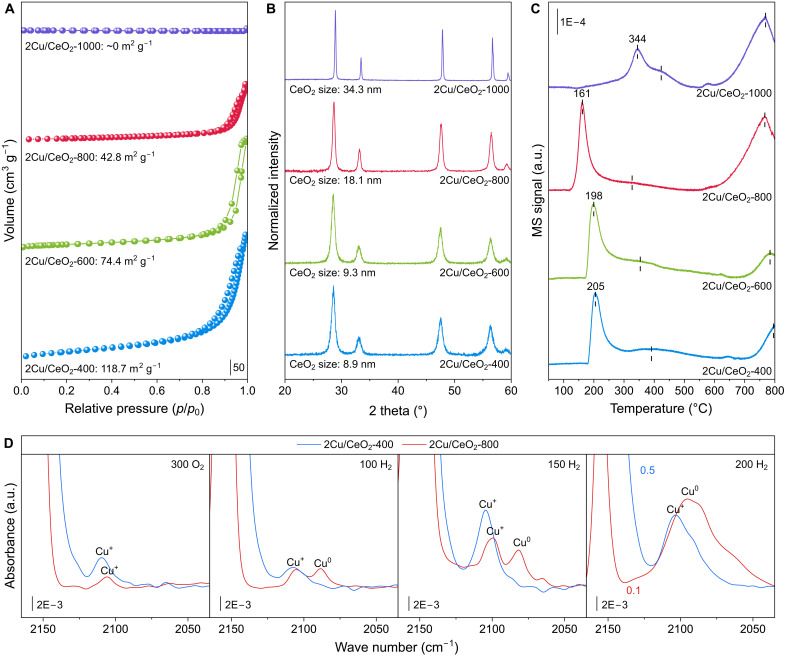
Thermal aging modulates the MSI in Cu/CeO_2_. (**A**) N_2_ sorption isotherms of 2Cu/CeO_2_-400, 2Cu/CeO_2_-600, 2Cu/CeO_2_-800, and 2Cu/CeO_2_-1000. The surface area was calculated via the BET method. (**B**) The XRD patterns of 2Cu/CeO_2_-400, 2Cu/CeO_2_-600, 2Cu/CeO_2_-800, and 2Cu/CeO_2_-1000. (**C**) H_2_-TPR profiles of 2Cu/CeO_2_-400, 2Cu/CeO_2_-600, 2Cu/CeO_2_-800, and 2Cu/CeO_2_-1000 with the mass spectrometry (MS) signal of H_2_O/He collected. (**D**) The in situ FTIR spectra of 2Cu/CeO_2_-800 and 2Cu/CeO_2_-400 after respective pretreatment under O_2_ at 300°C, H_2_ at 100°C, H_2_ at 150°C, and H_2_ at 200°C. a.u., arbitrary units.

Cu/CeO_2_ with 2 weight % (wt %) of Cu loading on CeO_2_ nanorods was synthesized via the deposition-precipitation method (see details in Materials and Methods) (*35*, *36*). The as-prepared sample was calcined at 400°, 600°, 800°, and 1000°C, and the resulting catalysts are denoted as 2Cu/CeO_2_-*T*, where *T* represents the calcination temperature. The Cu loading was verified by inductively coupled plasma atomic emission spectroscopy (ICP-AES). A series of characterizations was conducted to investigate the impact of calcination temperature on Cu/CeO_2_. Transmission electron microscopy (TEM) images revealed that both 2Cu/CeO_2_-400 and 2Cu/CeO_2_-600 retained the nanorod morphology of the CeO_2_ support, with widths of 10 to 20 nm and lengths of 100 to 200 nm (fig. S1). In contrast, after calcination at 800° or 1000°C, the CeO_2_ support sintered into irregular nanoparticles, with average sizes of about 25 nm for 2Cu/CeO_2_-800 and 70 nm for 2Cu/CeO_2_-1000. Isothermal N_2_ sorption further showed that the specific surface area of Cu/CeO_2_ catalysts decreased progressively with increasing calcination temperature. As shown in Fig. 1A, the Brunauer-Emmett-Teller (BET) surface area is 118.7 m^2^ g^−1^ for 2Cu/CeO_2_-400, 74.4 m^2^ g^−1^ for 2Cu/CeO_2_-600, and 42.8 m^2^ g^−1^ for 2Cu/CeO_2_-800. Notably, for 2Cu/CeO_2_-1000, the BET surface area becomes nearly negligible, indicating severe sintering of the CeO_2_ support. This trend is corroborated by x-ray diffraction (XRD) analysis (Fig. 1B), which shows a gradual increase in the CeO_2_ crystallite size from 8.9 nm for 2Cu/CeO_2_-400 to 34.3 nm for 2Cu/CeO_2_-1000. The above results demonstrate that thermal aging at temperatures above 800°C induces notable reconstruction and sintering of the CeO_2_ support.

H_2_ temperature-programmed reduction (H_2_-TPR) was performed to probe the MSI in these Cu/CeO_2_ catalysts. As shown in Fig. 1C, all TPR profiles display three distinct reduction features. The sharp low-temperature peak is attributed to the reduction of Cu species, which is followed by a broad intermediate-temperature peak associated with the reduction of strong Cu─O─Ce bonding (*33*, *35*). The high-temperature peak above 600°C corresponds to the bulk reduction of the CeO_2_ support. As the calcination temperature increases from 400° to 800°C, the reduction peak center of the Cu species shifts progressively lower, moving from 205° to 161°C. This shift is accompanied by a continuous decrease in the area of the intermediate-temperature peak, along with a decrease in overall H_2_ consumption (fig. S2). However, for 2Cu/CeO_2_-1000, the Cu reduction peak shifts to a much higher temperature of 344°C, a characteristic of bulk CuO. This result, together with N_2_ sorption isotherms (Fig. 1A), XRD patterns (Fig. 1B), and TEM images (fig. S1), suggests that excessive sintering of the CeO_2_ support at 1000°C leads to the formation of large CuO aggregates. Consequently, 800°C was identified as the optimal calcination temperature, mitigating the MSI while avoiding detrimental oversintering of the CeO_2_ support.

To further understand the effect of this optimal thermal aging treatment, we focused subsequent comparative experiments on the representative 2Cu/CeO_2_-400 and 2Cu/CeO_2_-800 samples. The CO-TPR was performed with profiles shown in fig. S3. It revealed that the reduction of Cu in both 2Cu/CeO_2_-400 and 2Cu/CeO_2_-800 by CO was more sensitive than that by H_2_. The onset reduction temperature of Cu in 2Cu/CeO_2_-800 (less than 50°C) was lower than in 2Cu/CeO_2_-400 (115°C). The CO-TPR profile of 2Cu/CeO_2_-400 shows much wider peaks, also suggesting a slower reduction of Cu. Moreover, 2Cu/CeO_2_-800 with ex situ prereduction under H_2_ at 200°C also exhibited pronounced reduction peaks, indicating a reoxidation in air. A N_2_O pulse was carried out on 2Cu/CeO_2_-400 and 2Cu/CeO_2_-800 after in situ H_2_ reduction at 200°C. The profile in fig. S4 revealed pronounced N_2_O consumption on prereduced 2Cu/CeO_2_-800, whereas negligible uptake was observed for prereduced 2Cu/CeO_2_-400. Using the selective N_2_O oxidation of Cu^0^ to Cu^+^, the Cu dispersion was quantified, from which the average Cu particle size was estimated to be 5.9 nm. It confirms the generation of abundant metallic Cu nanoparticles on 2Cu/CeO_2_-800 after H_2_ reduction at 200°C, while the Cu species in 2Cu/CeO_2_-400 remain cationic. Raman spectroscopy revealed a noticeable decrease in the intensity ratio of the defect-related *D* band to the *F*_2g_ vibration after thermal aging, indicating a reduction in oxygen vacancy concentration (fig. S5).

In situ Fourier transform infrared (FTIR) spectroscopy at 77 K was further performed with CO as a probe molecule to resolve the fine valence structure of Cu species in 2Cu/CeO_2_-400 and 2Cu/CeO_2_-800. A low-temperature measurement was necessary, since Cu^2+^ is easily reduced to Cu^+^ by CO even around room temperature, as demonstrated in the CO-TPR experiment (fig. S3). 2Cu/CeO_2_-800 and 2Cu/CeO_2_-400 were respectively in situ pretreated under O_2_ at 300°C, H_2_ at 100°C, H_2_ at 150°C, and H_2_ at 200°C for 30 min before cooling down to 77 K. The peaks above 2150 cm^−1^, within 2120 to 2100 cm^−1^, and below 2100 cm^−1^ were attributed to CO adsorbed on Ce^3+^, Cu^+^, and Cu^0^ sites, respectively (*37*, *38*). The CO adsorption on Cu^2+^ (>2150 cm^−1^) was weak and not distinguishable under experimental conditions. The full spectral range was shown in fig. S6, with the region of 2040 to 2160 cm^−1^ magnified and presented in Fig. 1D. After O_2_ pretreatment at 300°C, the spectra of both 2Cu/CeO_2_-800 and 2Cu/CeO_2_-400 showed only weak CO adsorption peaks above 2106 cm^−1^ corresponding to Cu^+^, indicating that Cu species remained oxidized. After H_2_ pretreatment at 100°C, the weak peak at 2088 cm^−1^ suggested partial reduction of Cu species to Cu^0^ in 2Cu/CeO_2_-800, whereas 2Cu/CeO_2_-400 only exhibited a peak at 2107 cm^−1^ assigned to Cu^+^. Following H_2_ pretreatment at 150°C, 2Cu/CeO_2_-800 exhibited a 6-cm^−1^ redshift in both Cu^0^ and Cu^+^ signals along with a slight intensity increase, proving further reduction of Cu. As for 2Cu/CeO_2_-400, enhanced intensity of Cu^+^ signal (2104 cm^−1^) was observed without any Cu^0^ features. After H_2_ pretreatment at 200°C, the Cu^0^ signal (2095 cm^−1^) in 2Cu/CeO_2_-800 increased about 20-fold, and the Cu^+^ signal disappeared, indicating extensive reduction of Cu species. In contrast, 2Cu/CeO_2_-400 showed only a twofold increase of Cu^+^ signal (2102 cm^−1^) with minor Cu^0^ signal below 2092 cm^−1^. The experiments above demonstrated that thermal aging substantially lowered the reduction temperature and increased the reduction degree of Cu species under H_2_, consistent with the prior TPR and N_2_O pulse results (Fig. 1C and fig. S4).

### The morphology of thermally aged Cu/CeO_2_

**Fig. 2.**
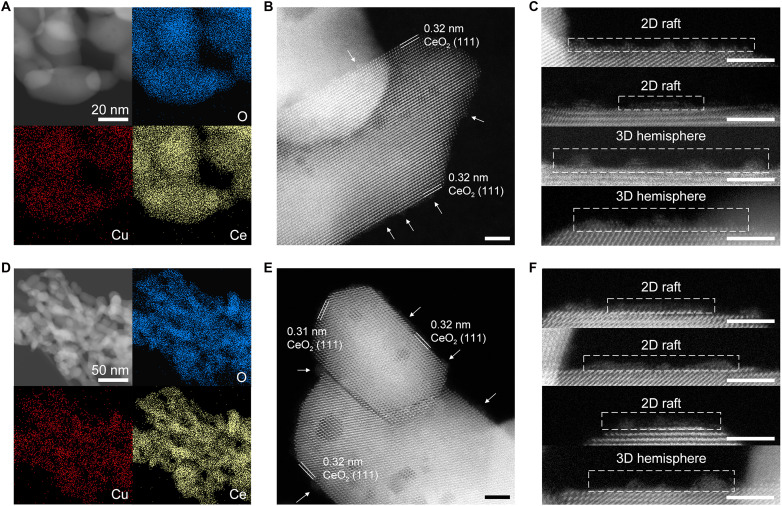
The morphology of thermally aged Cu/CeO_2_. (**A**) HRTEM image and EDS mapping of fresh 2Cu/CeO_2_-800. (**B**) HAADF-STEM image of fresh 2Cu/CeO_2_-800. (**C**) Magnified HAADF-STEM image of fresh 2Cu/CeO_2_-800 with the contrast adjusted to show the supported Cu nanoclusters clearly. (**D**) HRTEM image and EDS mapping of reduced 2Cu/CeO_2_-800. (**E**) HAADF-STEM images of reduced 2Cu/CeO_2_-800. (**F**) Magnified HAADF-STEM image of fresh 2Cu/CeO_2_-400 with the contrast adjusted to show the supported Cu nanoclusters clearly. The scale bar is 3 nm, unless marked in figures.

To elucidate the impact of mitigated MSI on the geometric structure of supported Cu, we took further microscopy characterizations. High-resolution transmission electron microscopy (HRTEM), energy dispersive spectroscopy mapping (EDS mapping), and high-angle annular dark-field scanning transmission electron microscopy (HAADF-STEM) were used to characterize the morphology of Cu in 2Cu/CeO_2_-800 before and after H_2_ reduction at 200°C. For fresh 2Cu/CeO_2_-800, no Cu particles are observed in HRTEM images and EDS mapping (Fig. 2A), suggesting the high dispersion of Cu. HAADF-STEM images show more detailed information about Cu morphology. As shown in Fig. 2B, the CeO_2_ support exposes (111) facets. Several few-atom Cu nanoclusters are observed on the CeO_2_ support, as pointed out by the arrows in the figure.

Because of the much lower contrast of Cu compared to Ce, supported Cu exhibits insufficient visibility when CeO_2_ regions are properly imaged in STEM. Therefore, we magnified some selected regions and adjusted their contrast accordingly, with the original STEM images provided in fig. S7. As shown in Fig. 2C, supported Cu exists as nanoclusters with a width below 2 nm, comprising both monolayer/bilayer two-dimensional (2D) rafts and few-atom-layered 3D hemispheres. This finding is consistent with the results of HRTEM and EDS mapping, indicating the high dispersion of Cu and potential catalytic applications of 2Cu/CeO_2_-800. We also performed HAADF-STEM characterization on the reference sample 2Cu/CeO_2_-400. As shown in fig. S8, the images indicate that the surface is covered with highly dispersed Cu clusters, exhibiting an extremely high density, which is distinctly different from 2Cu/CeO_2_-800. This difference indicates a stronger MSI in 2Cu/CeO_2_-400, resulting in high Cu coverage but low reducibility. Our thermal aging strategy mitigates the MSI, promoting moderate sintering of Cu species into more independent clusters that are easier to reduce.

For reduced 2Cu/CeO_2_-800, supported Cu species is also highly dispersed, as proved by HRTEM images and EDS mapping (Fig. 2D). STEM images reveal that the exposed facets are several (111) facets (Fig. 2E). The magnified STEM images also showed that Cu existed as nanoclusters with the shape of 2D rafts and 3D hemispheres (Fig. 2F and fig. S9). Similar microscopy results of fresh and reduced 2Cu/CeO_2_-800 suggest that H_2_ reduction seemingly does not influence the morphology of supported Cu. It should be noted that the reduced samples were inevitably exposed to air during transfer between reduction and HAADF-STEM imaging procedures. One noteworthy indicator is the reoxidation observed in the CO-TPR experiment (fig. S3), necessitating further in situ characterization of the potential impact of air exposure on the samples.

### The reversible structure evolution of Cu in thermally aged Cu/CeO_2_

**Fig. 3.**
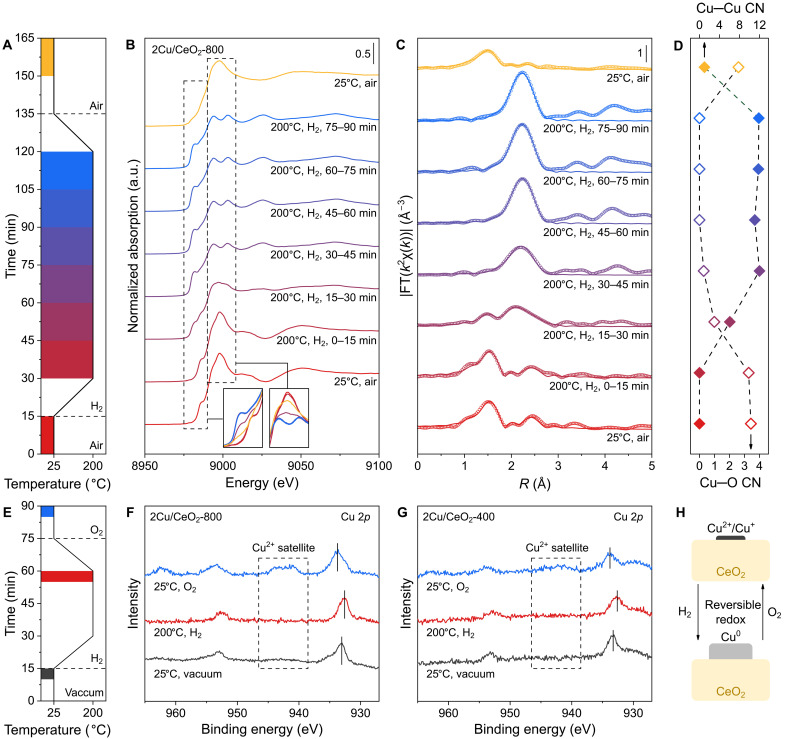
The reversible structure evolution of Cu in thermally aged Cu/CeO_2_. (**A**) The conditions of in situ XAS experiments. Pressure: 1 bar. (**B**) The in situ XANES spectra of 2Cu/CeO_2_-800 in the redox circle. (**C**) The EXAFS data of 2Cu/CeO_2_-800 in the redox circle. FT, Fourier Transform. (**D**) The CN of 2Cu/CeO_2_-800 derived from EXAFS data. (**E**) The conditions of in situ XPS experiments. Pressure: ultrahigh vacuum or 0.01 mbar of H_2_ or O_2_. (**F** and **G**) The Cu 2*p* XPS spectra of 2Cu/CeO_2_-800 (F) and 2Cu/CeO_2_-400 (G) in different atmospheres. (**H**) The scheme of reversible structure evolution of Cu/CeO_2_ under H_2_ and O_2_.

In situ x-ray absorption spectroscopy (XAS) was used to obtain in situ information on the chemical state and coordination of Cu in thermally aged Cu/CeO_2_. The spectra of 2Cu/CeO_2_-800 were collected in sequence under conditions of air at 25°C, H_2_ at 200°C with different reduction time, and air at 25°C (Fig. 3A). The Cu *K*-edge spectra of x-ray absorption near edge structure (XANES) were shown in Fig. 3B. In the air atmosphere at 25°C, the high intensity of the white peak and pre-edge signal pointed to the dominance of Cu^2+^. Under the H_2_ atmosphere at 200°C, the characteristic peak of Cu^0^ gradually appeared in the pre-edge region, while the white peak intensity decreased progressively. After 45 min of H_2_ treatment at 200°C, the characteristic signals of Cu^2+^ completely disappeared and remained nearly unchanged, indicating full reduction to Cu^0^. After H_2_ reduction, the sample was cooled down and exposed to air at 25°C. The white line and pre-edge peak intensity between the initial and reduced samples showed that Cu^0^ was partially reoxidized to a mixture of Cu^2+^ and Cu^+^.

Subsequently, we performed analysis and quantitative fitting on the extended x-ray absorption fine structure (EXAFS) data. As shown in Fig. 3 (C and D), fig. S10, and table S1, under an O_2_ atmosphere at 25°C, the first coordination of Cu exclusively consisted of O atoms, with Cu─O CN of 3.4. At 200°C under H_2_ atmosphere, the Cu─O coordination was progressively replaced by Cu─Cu coordination. During the reduction process within 45 min, the Cu─O CN gradually decreased from 3.3 to 1.0, while the Cu─Cu CN increased from 0.0 to 6.1. After 45 min of H_2_ reduction, Cu─O coordination was undetected, and the Cu─Cu CN remained in the range of 11.1 to 12.0, suggesting aggregation of Cu in H_2_. On the basis of the CN, the Cu particle size was estimated to exceed 6.2 nm (*39*), in agreement with the value derived from N_2_O chemisorption (fig. S4). After cooling to 25°C and air exposure, Cu─O coordination became dominant with a CN of 2.6, while the Cu─Cu CN decreased to 1.0. Notably, the sample reduced by H_2_ was rapidly reoxidized to a positive valence state upon reexposure to air at 25°C. These results demonstrate that in thermally aged Cu/CeO_2_, Cu species exist in an oxidized state under ambient air conditions and a metallic state under H_2_ at 200°C.

Near-ambient pressure x-ray photoelectron spectroscopy (NAP-XPS) was performed to probe the redox behavior of Cu/CeO_2_ in different atmospheres. 2Cu/CeO_2_-400 and 2Cu/CeO_2_-800 were respectively treated by vacuum at 25°C, H_2_ at 200°C, and O_2_ at 25°C (Fig. 3E). As shown in Fig. 3F, for 2Cu/CeO_2_-800, the Cu 2*p*_3/2_ peaks were at 933.0 eV (25°C, vacuum), 932.7 eV (200°C, H_2_), and 933.7 eV (25°C, O_2_), indicating H_2_ reduction followed by O_2_ reoxidation of Cu species. At the same time, the characteristic satellite peaks of Cu^2+^ (940 to 945 eV) disappeared under a H_2_ atmosphere and reappeared upon O_2_ exposure, confirming the reversible reduction and oxidation of Cu species. As shown in Fig. 3G, 2Cu/CeO_2_-400 demonstrated a similar redox behavior, with the Cu 2*p*_3/2_ peaks at 933.3 eV (25°C, vacuum) and 932.8 eV (200°C, H_2_), shifting to notably higher values of 933.8 eV (25°C, O_2_). Even considering the low pressure of NAP-XPS and the inherent slight difference in the Cu XPS peaks, the Cu 2*p*_3/2_ binding energy for 2Cu/CeO_2_-800 remains consistently 0.1 to 0.3 eV lower than that for 2Cu/CeO_2_-400 in all treatments. This shift supports the presence of Cu species in a lower oxidation state in 2Cu/CeO_2_-400 than that in 2Cu/CeO_2_-800, in line with the conclusions from in situ XAFS and FTIR (Figs. 1D and 3, B to D). On the basis of ex situ microscopy (Fig. 2, C and F) and in situ spectroscopy analysis (Fig. 3, A to F), we elucidate a reversible structural evolution of Cu species in Cu/CeO_2_, featuring a reversible redox and aggregation-dispersion behavior. Oxidized Cu nanoclusters aggregate into metallic nanoparticles with high Cu─Cu CN under H_2_ reduction and redisperse into partially oxidized nanoclusters upon air exposure at room temperature (Fig. 3H). Such redispersion phenomenon may be induced by reverse oxygen spillover mediated by MSI, wherein oxygen species from support CeO_2_ migrate onto Cu under oxidizing conditions (*26*, *40*, *41*).

The O 1*s* spectra of NAP-XPS for 2Cu/CeO_2_-400 and 2Cu/CeO_2_-800 were also collected and shown in fig. S11 (A and B). The sharp peaks at 529.7 eV and broad peaks around 531.7 eV were assigned to the lattice oxygen and hydroxyl oxygen, respectively. Fitting analysis revealed the highest ratio of hydroxyl oxygen to lattice oxygen in initial vacuum at 25°C, which progressively decreased in H_2_ reduction at 200°C and sequential O_2_ exposure at 25°C (fig. S11C). The atmosphere and temperature governed hydroxyl density, where reductive conditions favored hydroxyl generation via surface hydrogenation (*42*), and the elevated temperature promoted hydroxyl condensation and decomposition (*43*). Under an H_2_ atmosphere at 200°C, hydroxyl condensation predominated over surface hydrogenation, resulting in reduced hydroxyl density compared to the reference in vacuum at 25°C. Subsequent O_2_ introduction at 25°C induced oxidation of the hydroxyl that existed as Lewis acid sites (*44*), leading to additional hydroxyl decomposition. Furthermore, comparative analysis demonstrated lower hydroxyl density in 2Cu/CeO_2_-800 than in 2Cu/CeO_2_-400 under identical atmospheres. The diffuse reflectance infrared Fourier transform spectroscopy (DRIFTS) was further conducted with D_2_ introduction for surface hydroxyl exchange. As shown in fig. S12, the amount of hydroxyl signal on 2Cu/CeO_2_-800 was much lower than 2Cu/CeO_2_-400, aligning with NAP-XPS results (fig. S11). These results indicate that thermal aging effectively eliminates surface hydroxyl and concurrently suppresses their regeneration.

To check the influence of thermal aging on the CeO_2_ support, we characterized bare CeO_2_ calcined at 400° and 800°C (denoted as CeO_2_-400 and CeO_2_-800). HRTEM shows that CeO_2_-400 consists of nanorods with width of 10 to 20 nm and length of several hundred nm, whereas CeO_2_-800 forms irregular nanoparticles (fig. S13). The BET surface area decreases markedly from 93.1 m^2^ g^−1^ for CeO_2_-400 to only 3.5 m^2^ g^−1^ for CeO_2_-800 (fig. S14). Both values are notably lower than those of their Cu-loaded counterparts (118.7 m^2^ g^−1^ for 2Cu/CeO_2_-400 and 42.8 m^2^ g^−1^ for 2Cu/CeO_2_-800; Fig. 1A), indicating that the presence of Cu can mitigate the sintering of CeO_2_. Raman spectroscopy reveals a clear reduction in oxygen vacancy concentration for CeO_2_-800 compared to CeO_2_-400 (fig. S15). NH_3_-DRIFTS was performed to probe surface hydroxyl groups (fig. S16). The spectra show that after thermal aging at 800°C, the terminal hydroxyls decrease sharply while the bridged hydroxyls almost disappear. The hydroxyl signals are much weaker than those observed on 2Cu/CeO_2_-400 and 2Cu/CeO_2_-800, demonstrating that the presence of Cu alleviates the removal of surface hydroxyl groups under thermal aging.

To further isolate the effect of thermal aging on the support, we designed a cross-check experiment. A control sample, denoted as 2Cu/(CeO_2_-800)-400, was prepared by depositing 2 wt % Cu onto the CeO_2_-800, followed by air calcination at 400°C. H_2_-TPR was performed on 2Cu/(CeO_2_-800)-400, and its profile was compared with those of 2Cu/CeO_2_-800 and 2Cu/CeO_2_-400 (fig. S17). The 2Cu/(CeO_2_-800)-400 sample exhibited a high reduction peak of Cu at 271°C, indicating an even stronger MSI than 2Cu/CeO_2_-400. This finding can be rationalized through a consistent mechanism combined with the characterization of bare CeO_2_. The severe sintering and excessive removal of hydroxyl groups on the CeO_2_ support lead to an excessively weakened MSI, which, in turn, promotes the aggregation of Cu species. Consequently, the reduction profile shifts to resemble that of bulk CuO, aligning well with that observed for 2Cu/CeO_2_-1000.

Integrating these findings with previous literature, we can summarize the underlying mechanism for MSI modulation. As reported, surface hydroxyl groups on the oxide support serve as key anchoring sites for the initial stabilization of metal ions (*19*, *42*, *45*, *46*). In our Cu/CeO_2_ system, these hydroxyl groups can form strong Cu─O─Ce bonding through Cu^2+^ substitution for H^+^, which firmly anchors Cu species onto the support surface. Our systematic study, varying the temperature and sequence of thermal aging, validates that an excessively high hydroxyl content stabilizes Cu in an oxidation state, while an insufficient amount leads to Cu aggregation. The thermal aging after Cu loading achieves a moderate hydroxyl density for Cu anchoring, which consequently modulates the MSI. This optimal MSI enables the reduction as well as the mobility of Cu species to reversibly aggregate under H_2_ and redisperse upon O_2_ exposure. Our thermal aging strategy is further corroborated by our prior work, where thermal aging at 800°C was shown to weaken the MSI in Pt/CeO_2_, thus suppressing the redispersion of Pt clusters into less active single atoms (*45*). Moreover, this thermal aging strategy was successfully applied to mitigate the MSI in other CeO_2_-supported metal systems (fig. S18), confirming its general applicability as a complementary design paradigm to bulk composition engineering (*47*, *48*).

### The unlocked hydrogenation activity of thermally aged Cu/CeO_2_

**Fig. 4.**
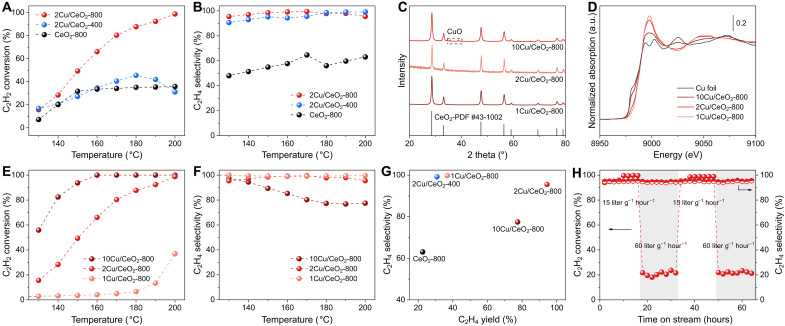
The unlocked hydrogenation activity of thermally aged Cu/CeO_2_. (**A** and **B**) The C_2_H_2_ conversion (A) and C_2_H_4_ selectivity (B) of 2Cu/CeO_2_-800, 2Cu/CeO_2_-400, and CeO_2_-800 toward acetylene semihydrogenation. (**C**) XRD patterns of 1Cu/CeO_2_-800, 2Cu/CeO_2_-800, and 10Cu/CeO_2_-800. (**D**) XANES spectra of Cu foil, 10Cu/CeO_2_-800, 2Cu/CeO_2_-800, and 1Cu/CeO_2_-800 after H_2_ reduction. (**E** and **F**) The C_2_H_2_ conversion (E) and C_2_H_4_ selectivity (F) of 10Cu/CeO_2_-800, 2Cu/CeO_2_-800, and 1Cu/CeO_2_-800 toward acetylene semihydrogenation. (**G**) The comparison of a series of Cu/CeO_2_ catalysts and CeO_2_ support on the catalytic performance in acetylene semihydrogenation. (**H**) The stability test of 2Cu/CeO_2_-800 at 200°C. Typical catalytic conditions: 1 bar, 0.5% C_2_H_2_, 5% H_2_, 50% C_2_H_4_, He balanced, 15 liter g^−1^ hour^−1^, unless marked in figure.

The H_2_-D_2_ exchange rate of 2Cu/CeO_2_-800 and 2Cu/CeO_2_-400 was measured to evaluate their intrinsic ability to activate H_2_. The H_2_-D_2_ exchange rate on 2Cu/CeO_2_-800 was faster than that on 2Cu/CeO_2_-400 in the range of 146° to 161°C (fig. S19). Meanwhile, the H_2_-D_2_ exchange reaction on 2Cu/CeO_2_-800 exhibited a lower apparent activation energy of 59.7 kJ mol^−1^ than that on 2Cu/CeO_2_-400 (66.2 kJ mol^−1^). The Cu/CeO_2_ catalysts were further tested in acetylene semihydrogenation, a reaction sensitive to the oxidation state and morphology of active sites in the catalyst (*25*, *49*, *50*). As shown in Fig. 4 (A and B), CeO_2_-800 exhibited poor catalytic performance with low C_2_H_2_ conversion (<40%) and C_2_H_4_ selectivity (<65%) at 130° to 200°C. Above 150°C, C_2_H_2_ conversion showed a negligible increase with temperature. 2Cu/CeO_2_-800 maintained a high selectivity (>95%) and continuously increasing conversion throughout the tested temperature range. It achieved C_2_H_2_ conversion of 98.8% and C_2_H_4_ selectivity of 95.5% at 200°C. In contrast, 2Cu/CeO_2_-400 exhibited a comparable C_2_H_2_ conversion (<45%) and much higher C_2_H_4_ selectivity than the bare CeO_2_ support. It was found that Cu species in 2Cu/CeO_2_-400 were nearly inactive in hydrogenation due to their high oxidation state, primarily serving as a modifier for enhancing C_2_H_4_ selectivity. Moreover, Raman spectroscopy of spent catalysts revealed carbon deposition on CeO_2_-800 and 2Cu/CeO_2_-400 but no carbon deposition on 2Cu/CeO_2_-800 (fig. S20). This confirms that the metallic Cu sites in 2Cu/CeO_2_-800 are crucial for efficient hydrogen activation, which suppresses coking and thus prevents the activity plateau observed at high temperatures (*47*, *51*–*53*). C_2_H_2_ temperature-programmed desorption (C_2_H_2_-TPD) shows that thermal aging has a minimal effect on acetylene activation (fig. S21), confirming that hydrogen activation is the primary factor for acetylene semihydrogenation performance.

Having established the unlocked hydrogenation activity of Cu/CeO_2_-800, the influence of Cu loading on structure and catalytic performance was further investigated. 1Cu/CeO_2_-800 and 10Cu/CeO_2_-800 with Cu loading of 1 and 10 wt % were prepared via a similar procedure to 2Cu/CeO_2_-800. As shown in Fig. 4C, only the XRD pattern of 10Cu/CeO_2_-800 showed an obvious peak of CuO, indicating the aggregation of Cu species. Excessive Cu loading in 10Cu/CeO_2_-800 induced the bulk aggregation of Cu, since the anchoring sites on the CeO_2_ support are insufficient. N_2_ sorption isotherms revealed a close surface area of 1Cu/CeO_2_-800, 2Cu/CeO_2_-800, and 10Cu/CeO_2_-800 (fig. S22). XPS spectra in fig. S23 found that the binding energies of the Cu 2*p*_3/2_ peaks for 1Cu/CeO_2_-800, 2Cu/CeO_2_-800, and 10Cu/CeO_2_-800 were 933.2, 933.0, and 932.8 eV, respectively. H_2_-TPR profiles also showed decreased reduction temperature with increased Cu loading (fig. S24). XANES spectra depicted a progressive decrease in white line intensities and increase in pre-edge peak intensities with increasing Cu loading in reduced 1Cu/CeO_2_-800, 2Cu/CeO_2_-800, and 10Cu/CeO_2_-800 (Fig. 4D). EXAFS analysis demonstrated that, after H_2_ reduction, Cu─O coordination was predominant in 1Cu/CeO_2_-800 and 2Cu/CeO_2_-800, whereas Cu─Cu coordination became predominant in 10Cu/CeO_2_-800 (figs. S25 and S26). The fitting of EXAFS spectra revealed the CN of Cu─O/Cu─Cu as 3.5/0.3 for 1Cu/CeO_2_-800, 2.6/1.0 for 2Cu/CeO_2_-800, and 1.5/6.2 for 10Cu/CeO_2_-800 (table S1). The results above indicated that increased Cu loading facilitated Cu reduction. The results of EXAFS and XRD demonstrated the formation of bulk Cu species in 10Cu/CeO_2_-800, which was absent in 1Cu/CeO_2_-800 and 2Cu/CeO_2_-800. Notably, although the state of Cu in Cu/CeO_2_ was distinct in in situ and ex situ conditions (Fig. 3), investigation of Cu loading effects on structure was achieved by comparing ex situ experiments with controlled conditions.

The catalytic performance of thermally aged Cu/CeO_2_ catalysts with varying Cu loadings toward the acetylene semihydrogenation was shown in Fig. 4 (E and F). 1Cu/CeO_2_-800 exhibited a low C_2_H_2_ conversion (<10%) at temperatures below 180°C and a rapid increase of C_2_H_2_ conversion only above 190°C. The low activity was assigned to the higher reduction temperature of Cu species, as evidenced by H_2_-TPR (fig. S24). On the other hand, the 10Cu/CeO_2_-800 with high Cu loading exhibited C_2_H_2_ conversion of 55.9% at 130°C and complete acetylene conversion at 160°C. However, 10Cu/CeO_2_-800 suffered from low C_2_H_4_ selectivity (<85%) when complete acetylene conversion was achieved. The accompanying strong hydrogenation activity thus improved hydrogenation activity at the expense of C_2_H_4_ selectivity. These results point to a structure-activity relationship where a lower valence state of Cu corresponds to higher hydrogenation activity, consistent with the reported literature (*54*). These loading-dependent structural trends reinforce that the moderate MSI is a key tunable parameter, which balances the conversion and selectivity of acetylene semihydrogenation.

The C_2_H_4_ selectivity and yield at 200°C of as-prepared Cu/CeO_2_ catalysts and CeO_2_ support were compared for a comprehensive evaluation of the catalytic performance (Fig. 4G). Thermally aged Cu/CeO_2_ with moderate loading of Cu, 2Cu/CeO_2_-800, appeared in the top-right region of the map, demonstrating simultaneous optimization of conversion (98.8%), selectivity (95.5%), and yield (94.4%). A stability test of 2Cu/CeO_2_-800 was performed at varying space velocities. As shown in Fig. 4H, both C_2_H_2_ conversion and C_2_H_4_ selectivity remained stable throughout the 60-hour continuous reaction, confirming the superior performance in sustained catalytic operation. The catalyst after the stability test was further characterized to examine the potential structural change. HRTEM images and EDS mapping show that Cu remains highly dispersed after reaction, and the CeO_2_ particle size remains largely unchanged compared to the reduced sample (fig. S27). Quasi–in situ XPS analysis confirms that Cu remains in the metallic state, consistent with its state after H_2_ reduction (fig. S28), demonstrating the structural stability of the catalyst. The results above confirm the successful modulation of active sites, endowing 2Cu/CeO_2_-800 with superior catalytic performance in acetylene semihydrogenation. It is noteworthy that, unlike reported catalyst design strategies such as constructing intermetallics (*55*–*58*), single-atomic isolation of Pd (*59*–*61*), and interfacial engineering (*62*–*64*), our approach unlocks the hydrogenation activity of inherently inactive Cu supported on CeO_2_ through MSI modulation, which releases Cu^0^ sites instead of the commonly formed Cu^+^ sites.

## DISCUSSION

In this work, we used a thermal aging strategy to modulate the MSI in Cu/CeO_2_ catalysts, facilitating the reduction and hydrogenation activity of the supported Cu while preserving the metal dispersion. TPR, CO-FTIR, and N_2_O pulse demonstrated that thermal aging lowered the reduction temperature and enhanced the reduction degree of Cu species in Cu/CeO_2_. HAADF-STEM results of 2Cu/CeO_2_-800 revealed that Cu remained in highly dispersed, few-atom-layered clusters in pre- and postreduction conditions. In situ XAS and NAP-XPS tracked a reversible structural evolution of Cu, where oxidized Cu nanoclusters aggregated into metallic nanoparticles with high CN in H_2_ and redispersed into partially oxidized nanoclusters upon air exposure at room temperature. The MSI modulation unlocked hydrogenation activity of Cu, yielding a substantially enhanced acetylene semihydrogenation performance compared to conventional counterparts. The present thermal treatment strategy offers a distinct path to modulate MSI with simplicity, compositional purity, and the ability to induce dynamic site reversibility.

## MATERIALS AND METHODS

### Chemicals

All the chemicals were of analytical grade and used without further purification. Ce(NO_3_)_3_·6H_2_O and H_2_PtCl_6_·6H_2_O were purchased from Aladdin. NaOH, Cu(NO_3_)_2_·3H_2_O, Ni(NO_3_)_2_·6H_2_O, Co(NO_3_)_2_·6H_2_O, Na_2_CO_3_, nitric acid, and H_2_O_2_ solution were purchased from Sinopharm Chemical Reagent Co. Ltd. All the gases were purchased from Air Liquide China. All the aqueous solutions were prepared using deionized water.

### Preparation of CeO_2_ support

The synthesis of CeO_2_ nanorods was conducted through a hydrothermal approach following optimized literature procedures (*65*). In a typical preparation, a solution was first prepared by dissolving 1.30 g of Ce(NO_3_)_3_·6H_2_O in 20 ml of deionized water. Simultaneously, a NaOH solution was obtained by dissolving 14.40 g of NaOH in 40 ml of deionized water. The cerium precursor solution was added to the NaOH solution under stirring for 30 min to form a homogeneous mixture. The resulting suspension was then transferred into a Teflon reactor and sealed within a stainless steel autoclave, followed by heating at 100°C for 24 hours. After cooling to ambient temperature, the precipitates were collected through centrifugation, followed by multiple rinsing cycles with deionized water to remove residual ions. The product was lastly dried at 60°C for 12 hours before the following application.

### Preparation of Cu/CeO_2_ catalysts

The supported catalysts were fabricated through an optimized deposition-precipitation technique adapted from established protocols (*33*, *34*). Typically, 0.5 g of CeO_2_ support was dispersed in 25 ml of deionized water for 30 min of stirring. A certain amount of Cu(NO_3_)_2_·3H_2_O was introduced into the slurry dropwise. The pH of this process was controlled at ~9 by adding Na_2_CO_3_ solution. The mixture was aged for 1 hour at ambient conditions to achieve precipitation equilibrium. The precipitates were filtered and washed with 1 liter of deionized water to eliminate ionic byproducts. Last, the product was dehydrated at 75°C for 12 hours, followed by calcination in a muffle furnace at 400° or 800°C.

### Preparation of other CeO_2_-supported catalysts

2Ni/CeO_2_-400, 2Ni/CeO_2_-800, 2Co/CeO_2_-400, 2Co/CeO_2_-800, 1Ni1Cu/CeO_2_-400, and 1Ni1Cu/CeO_2_-800 were synthesized following a similar method of preparing Cu/CeO_2_ catalysts. A total of 0.5 g of CeO_2_ support was dispersed in 25 ml of deionized water for 30 min of stirring. For Ni/CeO_2_ catalysts, a certain amount of Ni(NO_3_)_2_·6H_2_O was introduced into the slurry dropwise. For Co/CeO_2_ catalysts, a certain amount of Co(NO_3_)_2_·6H_2_O was introduced into the slurry dropwise. For NiCu/CeO_2_ catalysts, a certain amount of Ni(NO_3_)_2_·6H_2_O and Cu(NO_3_)_2_·3H_2_O was introduced into the slurry dropwise. The pH of this process was controlled at ~9 by adding Na_2_CO_3_ solution. The mixture was aged for 1 hour at ambient conditions to achieve precipitation equilibrium. The precipitates were filtered and washed with 1 liter of deionized water to eliminate ionic byproducts. Last, the product was dehydrated at 75°C for 12 hours, followed by calcination in a muffle furnace at 400° or 800°C.

0.5Pt/CeO_2_-400 and 0.5Pt/CeO_2_-800 were synthesized via the wet impregnation method. Typically, a certain amount of aqueous H_2_PtCl_6_·6H_2_O solution was mixed with 0.5 g of CeO_2_ support in 40 ml of deionized water. After stirring for 2 hours, the sample was collected in a rotary evaporator at 60°C. The product was then dehydrated at 75°C for 12 hours, followed by calcination in a muffle furnace at 400° or 800°C.

### Temperature-programmed reduction

The H_2_-TPR and CO-TPR experiments were performed on a VODO (Quzhou) chemisorption instrument with outlet gas analyzed by a mass spectrometer. In H_2_-TPR, 100 mg of the sample was first pretreated by He at 300°C for 1 hour, followed by cooling down to 50°C. Afterward, the gas was switched to 10% H_2_/Ar (30 ml min^−1^), and the experiment was measured from 50° to 800°C with a ramp rate of 10°C min^−1^. In CO-TPR, 100 mg of the sample was first pretreated by He at 300°C for 1 hour, followed by cooling down to 50°C. Afterward, the gas was switched to 10% CO/Ar (30 ml min^−1^), and the experiment was measured from 50° to 400°C with a ramp rate of 10°C min^−1^.

### In situ FTIR experiments

In situ FTIR experiments were performed on a JASCO FT/IR-4600 instrument fitted with a custom transmission cell featuring CaF_2_ windows. The sample was compressed into a self-supporting wafer of ~60 mg, followed by being affixed to a holder. Samples were in situ pretreated under corresponding conditions for 30 min. Sequentially, the cell was cooled down to 77 K in liquid nitrogen, with the background spectra collected in He. The spectra were then collected after introducing 10 mbar of CO.

### In situ XAS experiments

XAS measurements were conducted under fluorescence mode at the BL11B beamline of the Shanghai Synchrotron Radiation Facility (SSRF). The sample was fixed in a laboratory-made cell with gas controlled by mass flow controllers. The absorption-edge energies of the Cu foil were calibrated to the standard value. Athena and Artemis codes were used to analyze the EXAFS data. Parameters such as CN, bond distance, and Debye-Waller factor around the absorbed atoms were allowed to vary during the fitting.

### NAP-XPS experiments

NAP-XPS experiments were performed at the BL02B01 beamline of the SSRF. An incident photon energy of 1486 eV was used. The pressure of the chamber was controlled within 0.1 mbar. The data was fitted on XPSpeak software.

### Microscopy analysis

TEM images were taken using a Hitachi H-7700 transmission electron microscope at an acceleration voltage of 100 kV. HRTEM and EDS mapping were performed on a JEOL F200 field-emission transmission electron microscope operating at 200-kV accelerating voltage. HAADF-STEM images were collected on a Thermo Fisher Scientific Themis Z (3.2) field-emission transmission electron microscope. The reduced samples were inevitably exposed to air before HAADF-STEM or HRTEM imaging.

### N_2_O pulse

N_2_O pulse was performed using BELCAT II (MicrotracBEL) connected to a mass spectrometer (BELMass, MicrotracBEL Corporation). The amount of injected N_2_O was quantified using a pulse-quantification loop. One hundred milligrams of sample was pretreated by 10% H_2_/Ar at 200°C for 30 min and cooled to 50°C, followed by N_2_O pulses.

N_2_O is highly selective at 50°C and reacts only with the exposed metallic Cu atoms via the following equation

$$N_{2}O+\text{2Cu}=N_{2}+{\text{Cu}}_{2}O$$ (1)

Therefore, we calculate the Cu dispersion (*D*) as

$$D=n_{\text{Cu surface}}/n_{\text{Cu}}=\left(2\times n_{N2O}\right)/\left(m\times X/M\right)$$ (2)

where *n*_N2O_ is the moles of N_2_O consumption, *m* is the sample mass, *X* is the weight loading of Cu in the sample, and *M* is the atomic weight of Cu.

The Cu particle size (*d*) was then estimated from the dispersion. By modeling Cu as hemispheres, the relationship between dispersion and particle size can be derived as

$$D=n_{\text{Cu surface}}/n_{\text{Cu}}=7\times M/\left(a_{\text{Cu}─\text{Cu}}\times \rho \times N_{A}\times d\right)$$ (3)

where *a*_Cu─Cu_ is the Cu─Cu bond length, ρ is the density of metallic Cu, and *N*_A_ is Avogadro’s number. Substituting the values, we obtain

d=1.26/D (4)

### Diffuse reflectance infrared Fourier transform spectroscopy

The DRIFTS spectra were collected using a Bruker Vertex 70 FTIR spectrometer with a mercury cadmium telluride detector cooled by liquid nitrogen. For H_2_-D_2_ DRIFTS, before the measurement, the sample was pretreated in 10% H_2_/Ar gas at 200°C for 1 hour. The background spectra were collected until a stable baseline was reached. Then, 10% D_2_/Ar gas was introduced into the cell, followed by continuous recording of spectra maintained for 20 min. For NH_3_-DRIFTS, before the measurement, the sample was pretreated in He at 50°C for 15 min. The background spectra were collected until a stable baseline was reached. Then, 10% NH_3_/Ar gas was introduced into the cell, followed by continuous recording of spectra maintained for 20 min.

### Instrumentations

XRD patterns were recorded using a Philips X’Pert Pro Super diffractometer with Cu-Kα radiation (λ = 1.54178 Å). The scan rate was set to 3°/min. The crystallite size (*L*) of the catalyst was calculated by using the following Scherrer equation

L=K×λ/(β×cosθ) (5)

where λ is the wavelength of the x-ray radiation, θ is the diffraction angle, *K* is a constant taken as 0.89, and β is the full width at half maximum.

ICP-AES (Atomscan Advantage, Thermo Jarrell Ash, USA) was used to determine the loading of Cu. The samples were digested by a solution of 30% H_2_O_2_ and 70% nitric acid in a 1:1 ratio at room temperature for 2 hours. The resulting solution was diluted to an appropriate concentration to be analyzed.

The N_2_ sorption isotherms were measured on a Builder SSA-4200 surface area analyzer at 77 K after vacuum activation. Raman spectra were obtained by excitation of the samples with a 532-nm laser, using a LabRAM HR Evolution Raman spectrometer.

XPS measurements were conducted on an ESCALAB 250 (Thermo-VG Scientific, USA) with an Al Kα x-ray source (1486.6-eV photons) in constant analyzer energy mode with a pass energy of 30 eV for all spectra. For quasi–in situ XAFS, samples were pretreated in a tube furnace, followed by sealing up at room temperature and transferring into a glovebox. Subsequently, samples were sealed in a cell to keep air uncontacted.

C_2_H_2_-TPD was performed on a VODO (Quzhou) chemisorption instrument with outlet gas analyzed by a mass spectrometer. One hundred milligrams of the sample was first pretreated by H_2_ at 200°C for 1 hour, followed by cooling down to 50°C. Afterward, the gas was switched to 10% C_2_H_2_/Ar (30 ml min^−1^), and the experiment was measured from 50° to 200°C with a ramp rate of 10°C min^−1^.

### Catalytic test

The H_2_-D_2_ exchange was conducted on a fixed bed with the outlet detected by the mass spectrometer. The catalyst was prereduced by 10% H_2_/Ar at 200°C, followed by the introduction of 10% H_2_/10% D_2_/Ar.

The acetylene semihydrogenation was carried out in a continuous-flow fixed-bed reactor at atmospheric pressure. The gas flow was controlled by several mass flow controllers, and the temperature of the catalyst bed was monitored by a thermal couple at the catalyst bed. In a typical test, 50 mg of catalyst was diluted with 1 g of quartz (40 to 60 mesh). The catalyst was prereduced by 10% H_2_/Ar at 200°C. The gas mixture contained 0.5 vol % C_2_H_2_, 5 vol % H_2_, 50 vol % C_2_H_4_, and balanced He. The flow rate was 12.5 ml min^−1^. The reactants and products were analyzed by gas chromatography at each temperature after stabilization for 20 min with a flame ionization detector. C_2_H_2_ conversion (*X*) and C_2_H_4_ selectivity (*S*) were calculated using the following equations

$$\text{X}=100\%\times \left(C_{C2\text{H2 in}}-C_{C2\text{H2 out}}\right)/C_{C2\text{H2 in}}$$ (6)

$$\begin{array}{cc} S & =100\%\times \left(C_{C2\text{H2 in}}-C_{C2\text{H2 out}}\right)/\left(C_{C2\text{H2 in}}\right.\\ & \left.-C_{C2\text{H2 out}}+C_{C2\text{H6 out}}+C_{C4Hx\;\text{out}}\right) \end{array}$$ (7)

where *C*_C2H2 in_, *C*_C2H2 out_, *C*_C2H6 out_, and *C*_C4H*x* out_ were concentration of C_2_H_2_ at the inlet, C_2_H_2_, C_2_H_6_, and C_4_H*_x_* at the outlet, respectively.
