# Supplementary material for: Reversible aggregation-redispersion of Cu sites in Cu/CeO2 catalysts with unlocked hydrogenation activity
Source: Sci Adv. 2026 Mar 27;12(13):eaed2774. doi: 10.1126/sciadv.aed2774 (PMC13025107; doi:10.1126/sciadv.aed2774)
Supplement: Supplementary file 1 — Figs. S1 to S28 Table S1 [file sciadv.aed2774_sm.pdf]

Science Advances
AAAS

Supplementary Materials for

Reversible aggregation-redispersion of Cu sites in Cu/CeO_2 catalysts with unlocked hydrogenation activity

Yu Zhang *et al.*

Corresponding author: Han Yan, hanyanustc@ustc.edu.cn; Chun-Jiang Jia, jiacj@sdu.edu.cn; Jie Zeng, zengj@ustc.edu.cn

Sci. Adv. **12**, eaed2774 (2026)
DOI: 10.1126/sciadv.aed2774

This PDF file includes:

Figs. S1 to S28
Table S1

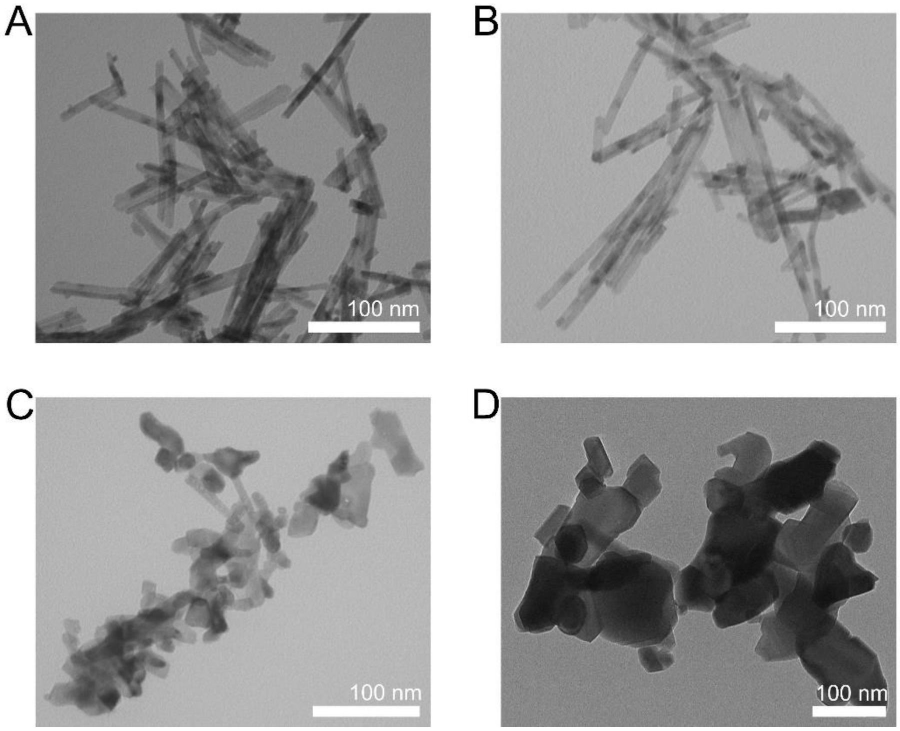

Fig. S1. TEM imaging. (**A** to **D**) TEM images of 2Cu/CeO_2-400 (A), 2Cu/CeO_2-600 (B), 2Cu/CeO_2-800 (C), and 2Cu/CeO_2-1000 (D).

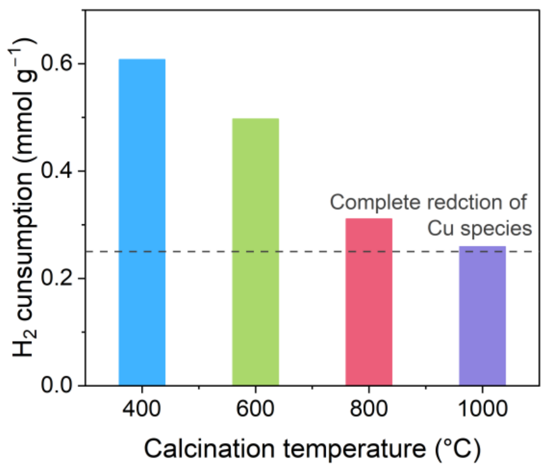

Fig. S2. Quantitative analysis of H_2-TPR experiment. The H_2 consumption of H_2-TPR profiles of 2Cu/CeO_2-400, 2Cu/CeO_2-600, 2Cu/CeO_2-800, and 2Cu/CeO_2-1000. The calculated consumption excludes the bulk reduction of CeO_2 support.

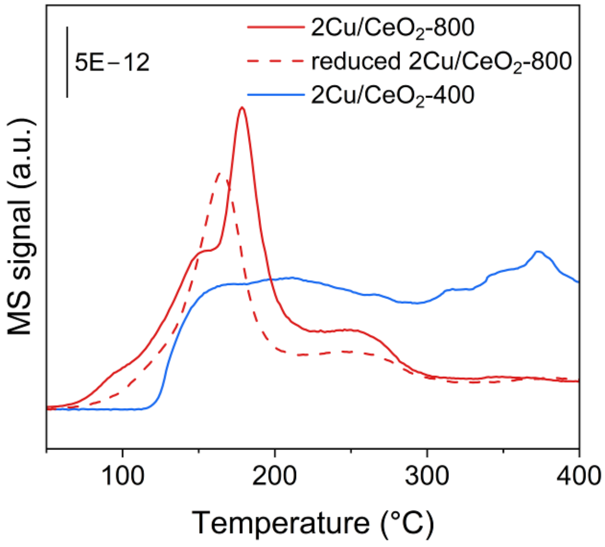

Fig. S3. CO-TPR characterization. The CO-TPR profiles of fresh $2Cu/CeO_2$-800, reduced $2Cu/CeO_2$-800, and fresh $2Cu/CeO_2$-400 with the CO_2 signal collected.

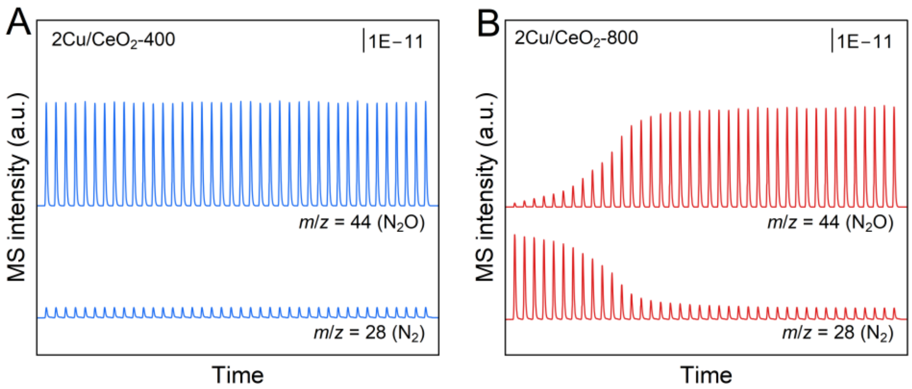

Fig. S4. N_2O-pulse characterization. (**A** and **B**) The N_2O-pulse profiles of 2Cu/CeO_2-800 (A) and 2Cu/CeO_2-400 (B) after H_2 reduction at 200 °C.

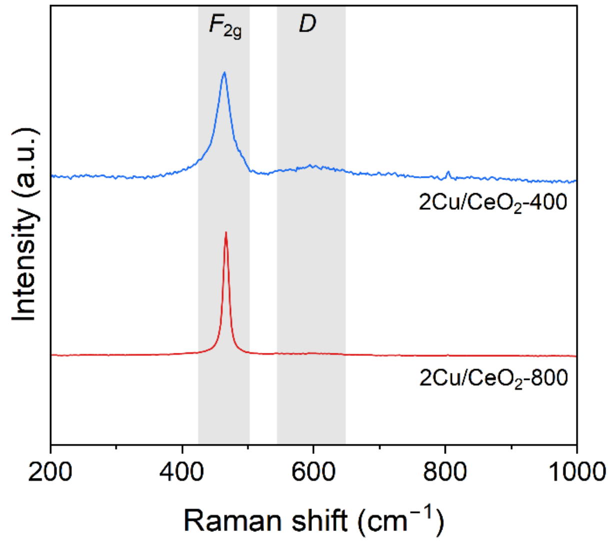

Fig. S5. Raman characterization. Raman spectra of 2Cu/CeO_2-800 and 2Cu/CeO_2-400.

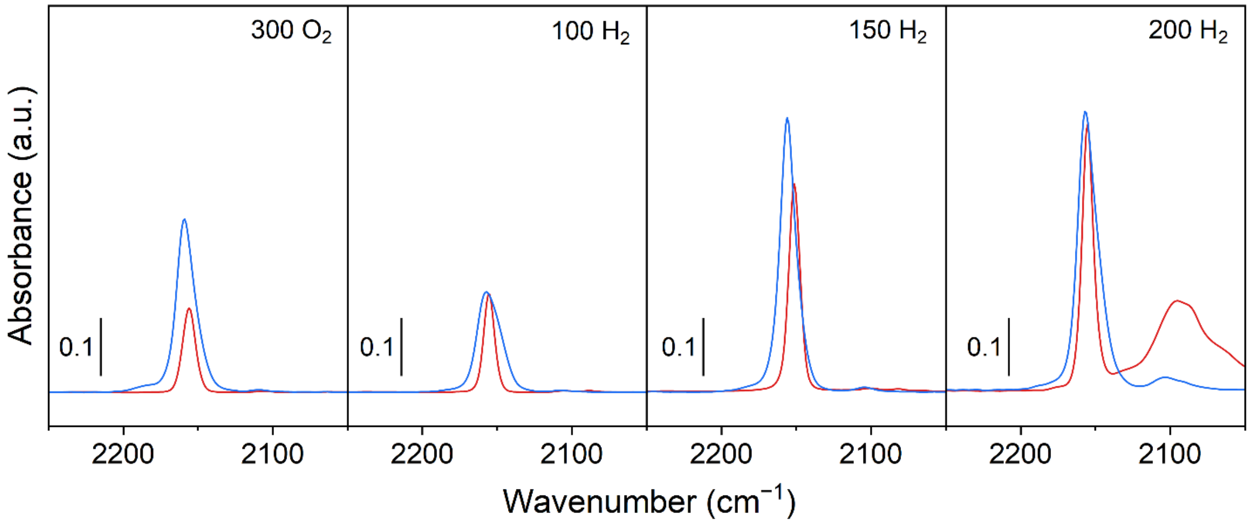

Fig. S6. *In-situ* FTIR characterization. The *in-situ* FTIR spectra of 2Cu/CeO_2-800 and 2Cu/CeO_2-400 after respectively pretreated under O_2 at 300 °C, H_2 at 100 °C, H_2 at 150 °C, and H_2 at 200 °C.

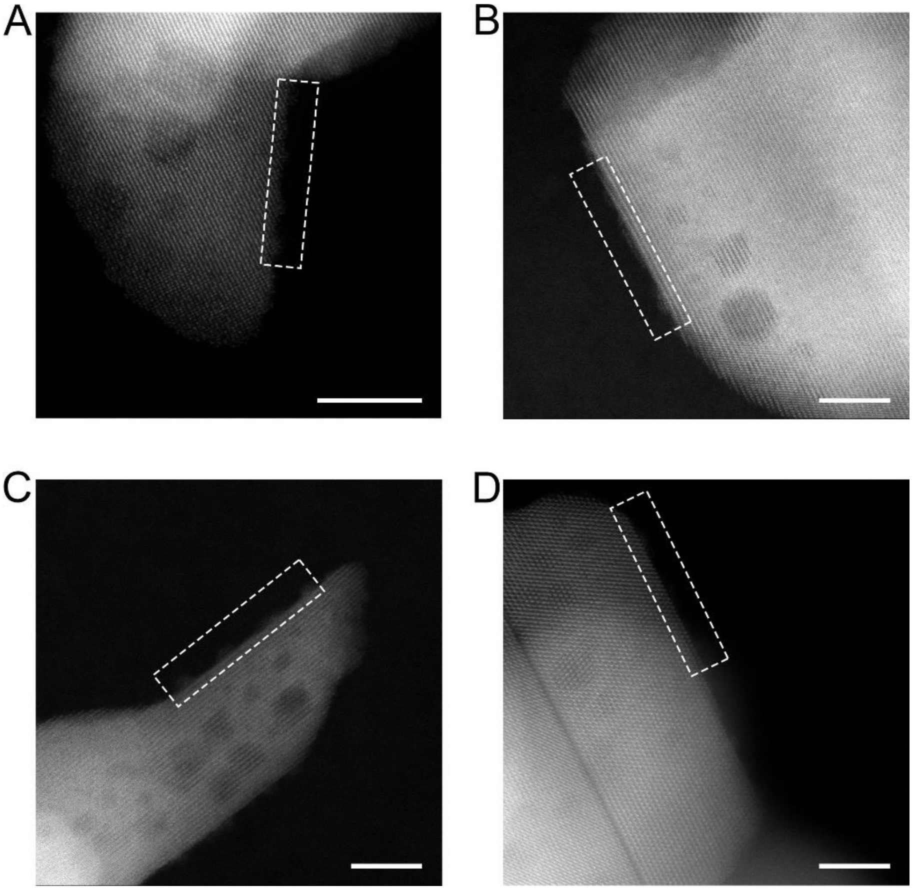

Fig. S7. HAADF-STEM imaging of fresh 2Cu/CeO_2-800. (**A** to **D**) HAADF-STEM images of fresh 2Cu/CeO_2-800. The scale bar is 5 nm. The square regions were magnified and shown in Fig. 2C with their contrast adjusted.

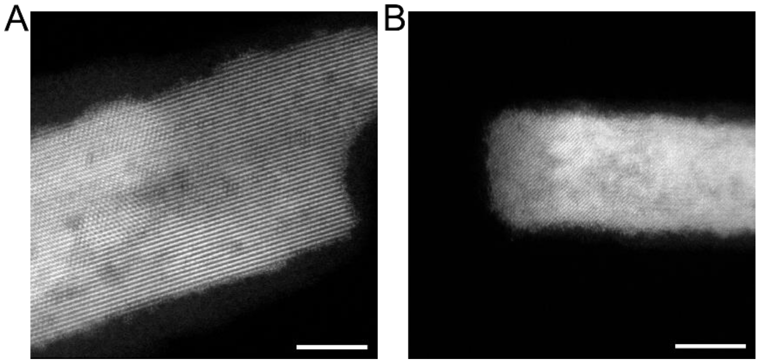

Fig. S8. HAADF-STEM imaging of 2Cu/CeO_2-400. (**A** and **B**) The HAADF-STEM images of 2Cu/CeO_2-400. The scale bar is 5 nm.

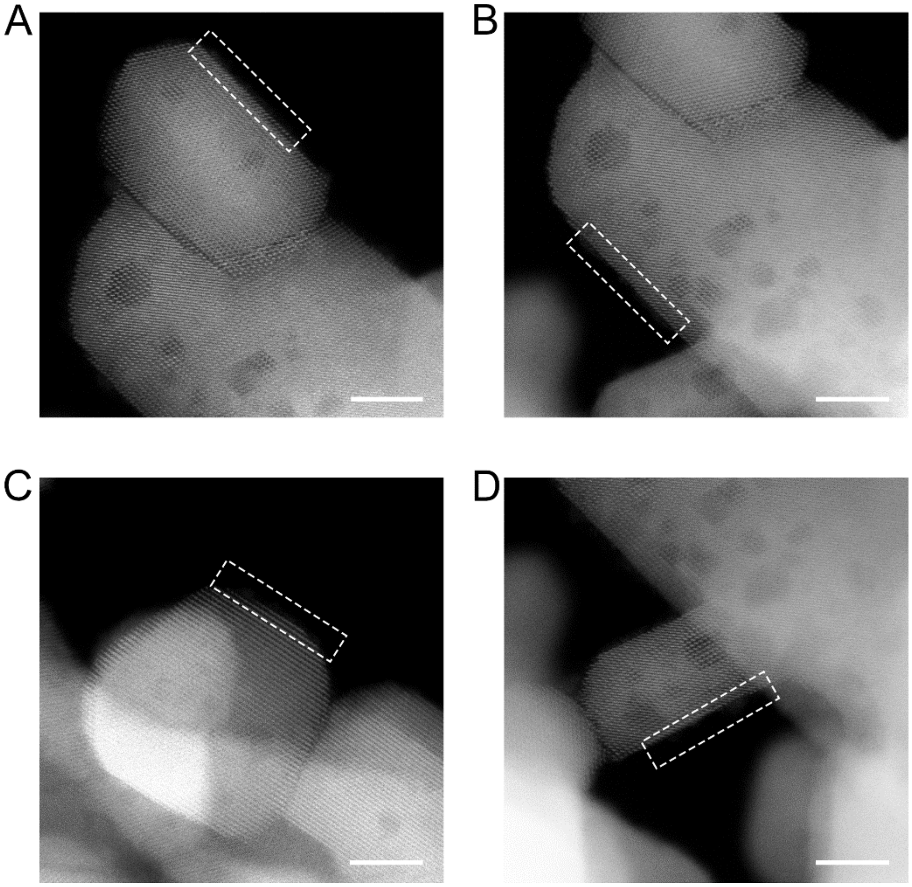

Fig. S9. HAADF-STEM imaging of reduced 2Cu/CeO_2-800. (**A** to **D**) HAADF-STEM images of reduced 2Cu/CeO_2-800. The scale bar is 5 nm. The square regions were magnified and shown in Fig. 2F with their contrast adjusted.

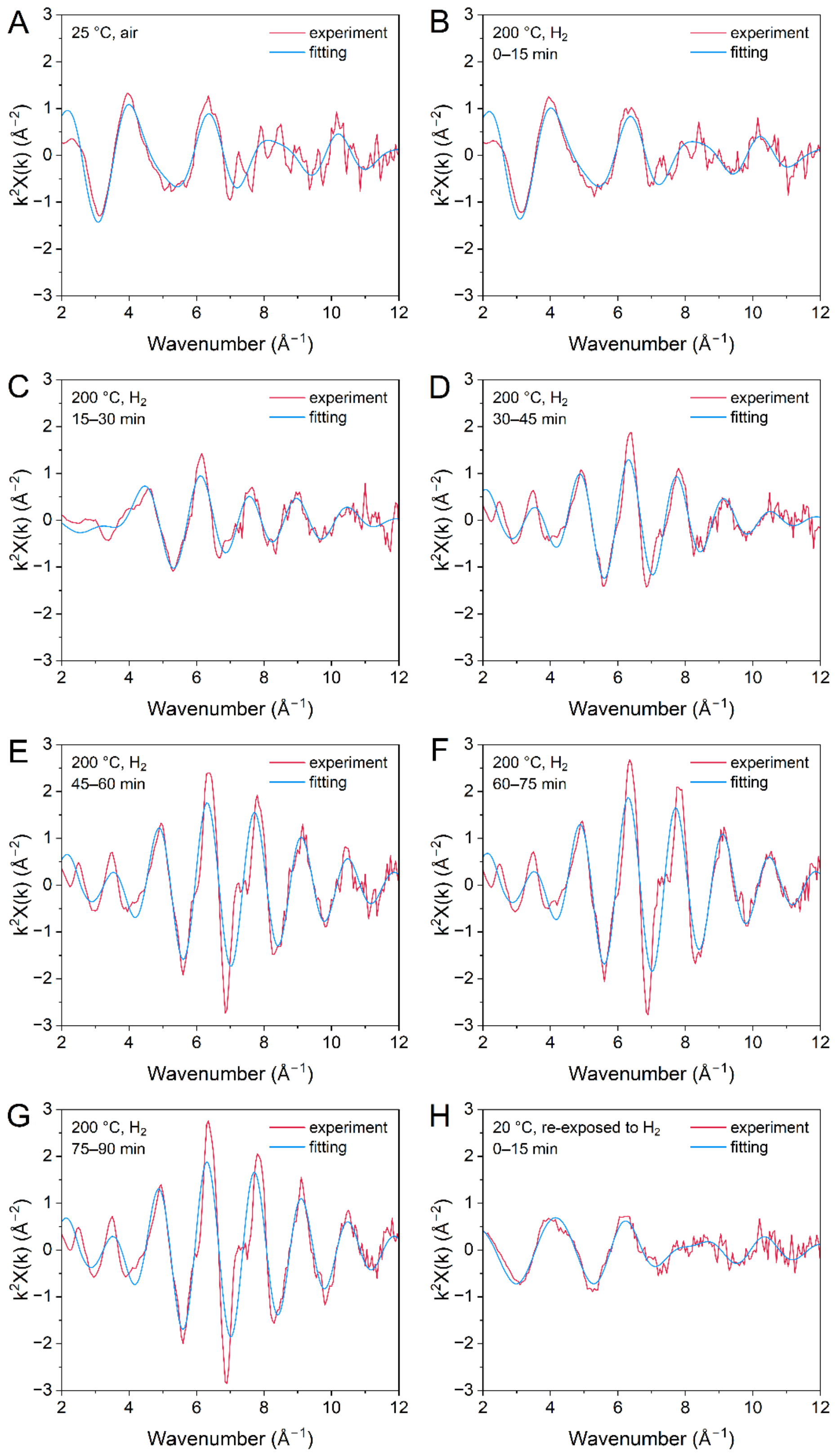

Fig. S10. Additional information on *in-situ* EXAFS. (**A** to **H**) The *k*-range profiles of *in-situ* EXAFS of 2Cu/CeO_2-800.

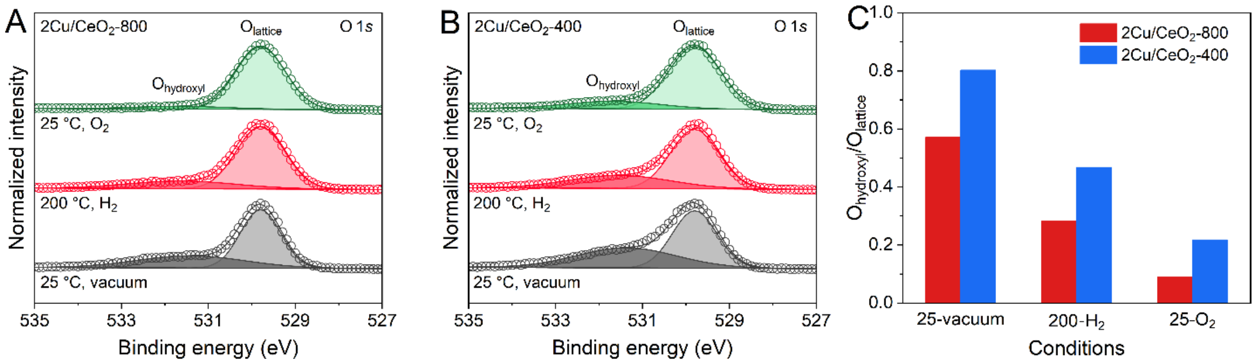

Fig. S11. The characterization of surface hydroxyl in NAP-XPS. (**A** and **B**) The O 1*s* spectra of NAP-XPS for 2Cu/CeO_2-800 (A) and 2Cu/CeO_2-400 (B) under different atmospheres. The detailed conditions were shown in Fig. 3E. (**C**) The hydroxyl oxygen to lattice oxygen ratio in 2Cu/CeO_2-800 and 2Cu/CeO_2-400 under various conditions derived from XPS fitting.

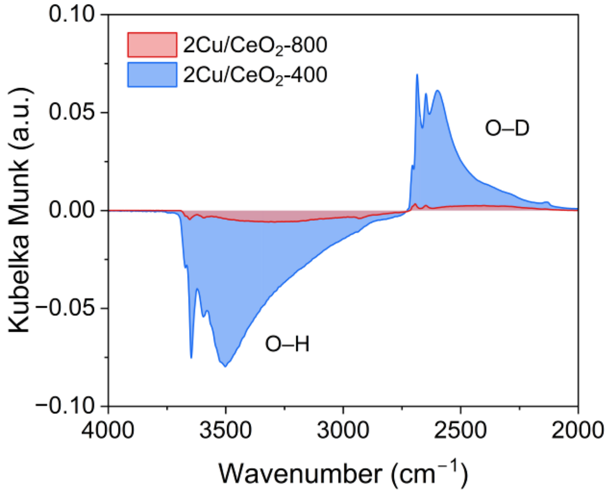

Fig. S12. The characterization of surface hydroxyl by DRIFTS. The DRIFTS spectra of $2Cu/CeO_2$-800 and $2Cu/CeO_2$-400 after introducing D_2. Samples were pretreated at 200 °C by H_2 reduction. The background was then collected in He at 200 °C.

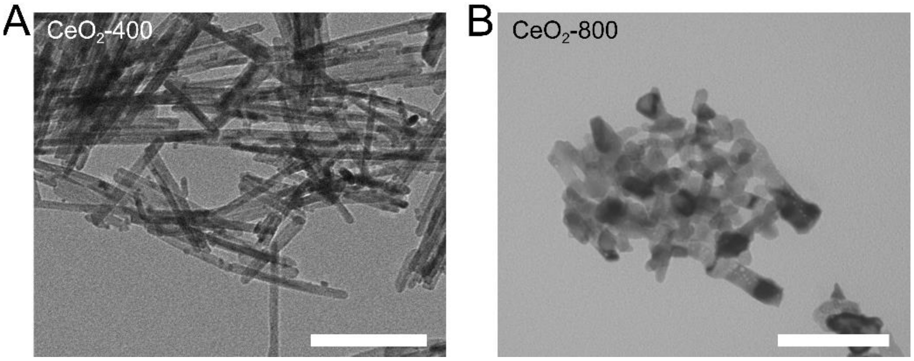

Fig. S13. TEM imaging of pure CeO_2. (**A** and **B**) TEM images of CeO_2-400 (A) and CeO_2-800 (B). The scale bar is 100 nm.

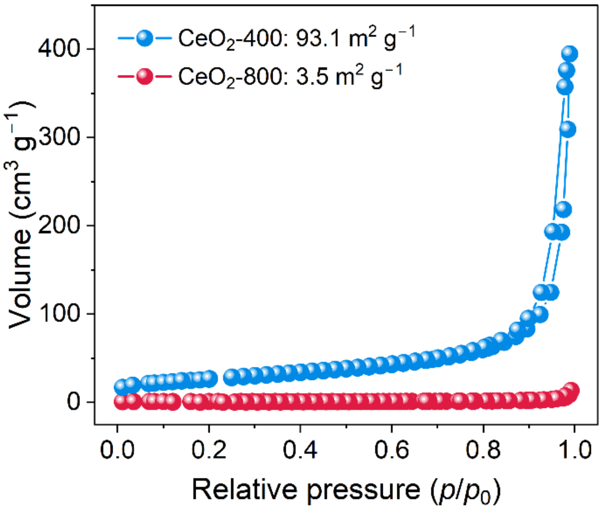

Fig. S14. Surface area characterization of pure CeO_2. N_2 sorption isotherms of CeO_2-400 and CeO_2-800. The surface area was calculated via the BET method.

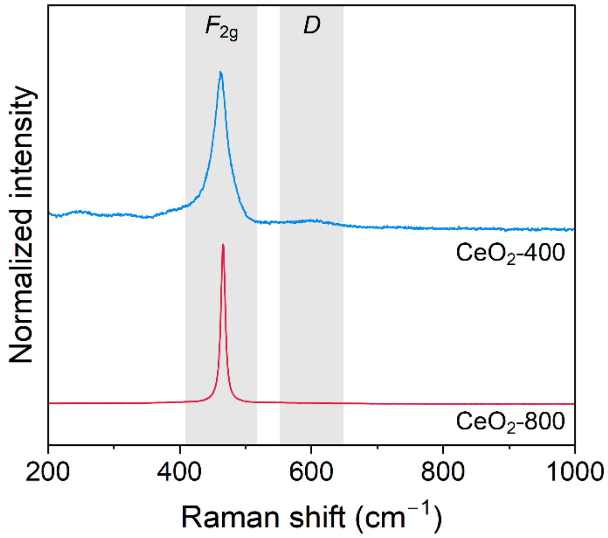

Fig. S15. Raman characterization of pure CeO_2. Raman spectra of CeO_2-400 and CeO_2-800.

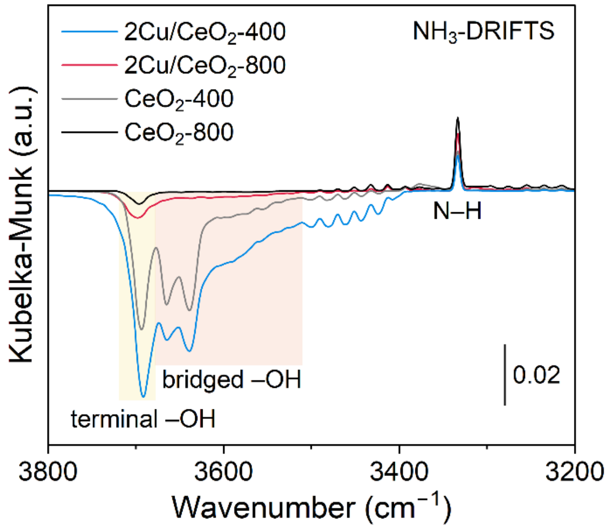

Fig. S16. Hydroxyl characterization by DRIFTS. NH_3-DRIFTS spectra of 2Cu/CeO_2-400, 2Cu/CeO_2-800, CeO_2-400, and CeO_2-800.

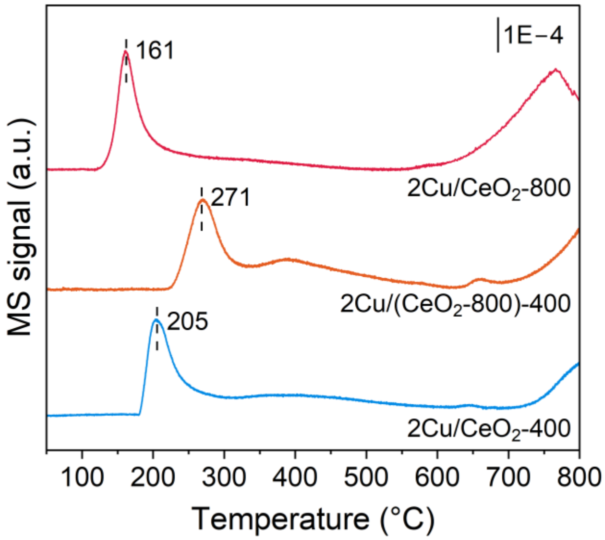

Fig. S17. Cross-check experiment. H_2-TPR profiles of 2Cu/CeO_2-400, 2Cu/(CeO_2-800)-400, 2Cu/CeO_2-800 with the H_2O/He signal collected.

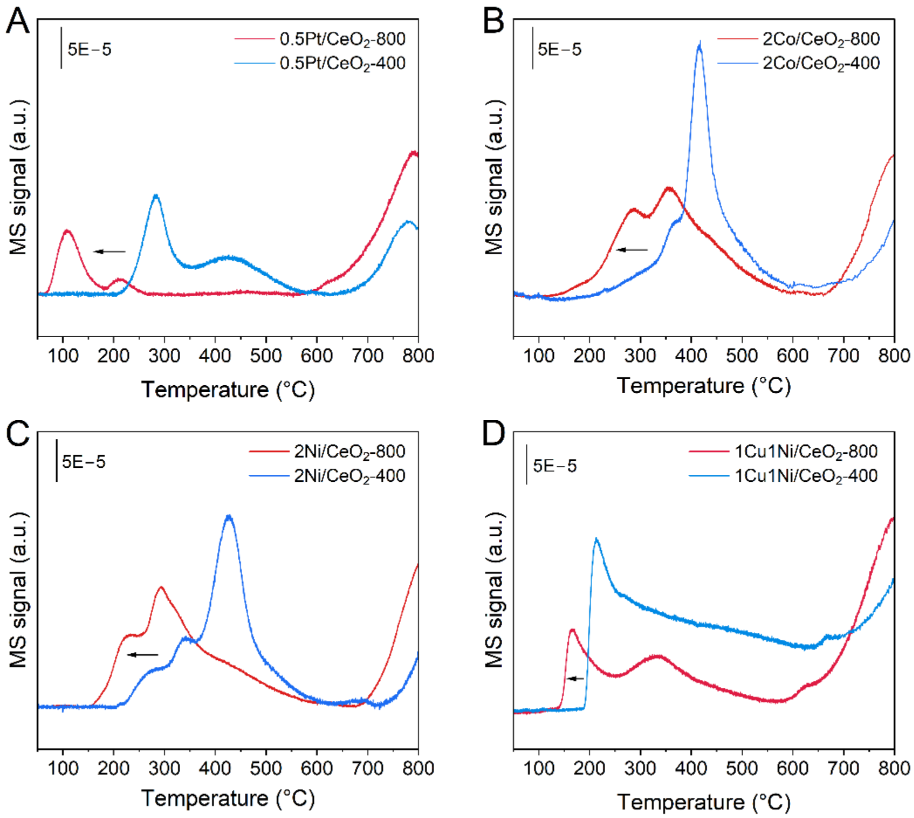

Fig. S18. Universality of the thermal aging strategy. (A to **D**) H_2-TPR profiles of 0.5Pt/CeO_2-400 and 0.5Pt/CeO_2-800 (A), 2Co/CeO_2-400 and 2Co/CeO_2-800 (B), 2Ni/CeO_2-400 and 2Ni/CeO_2-800 (C), and 1Ni1Cu/CeO_2-400 and 1Ni1Cu/CeO_2-800 (D) with the H_2O/He signal collected.

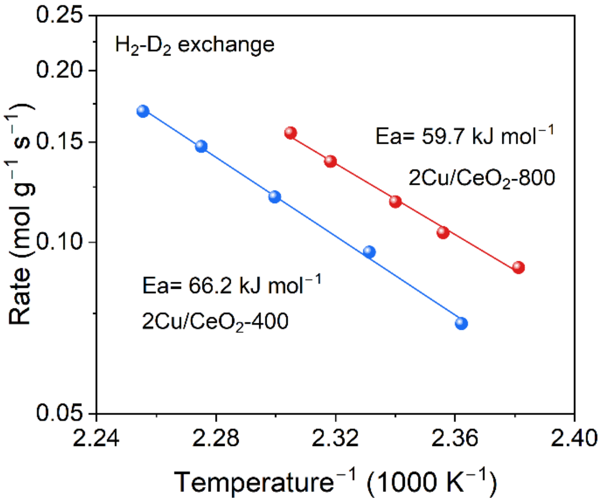

Fig. S19. H_2-D_2 exchange test. The test of the H_2-D_2 exchange rate on 2Cu/CeO_2-800 and 2Cu/CeO_2-400.

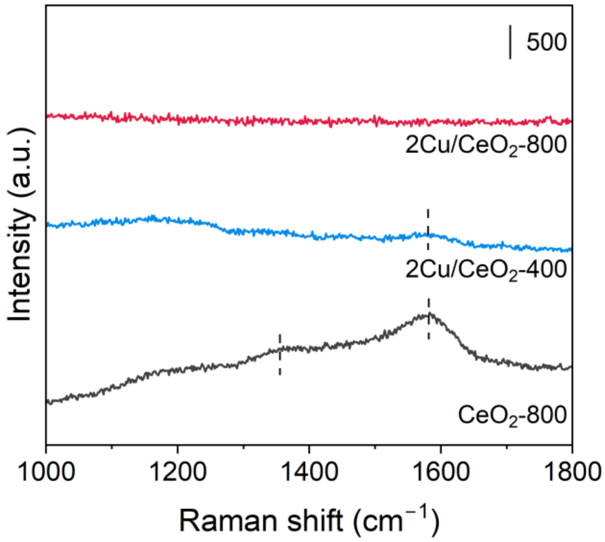

Fig. S20. Characterization of coke. The Raman spectra of 2Cu/CeO_2-800, 2Cu/CeO_2-400, and CeO_2-800 after acetylene semi-hydrogenation reaction.

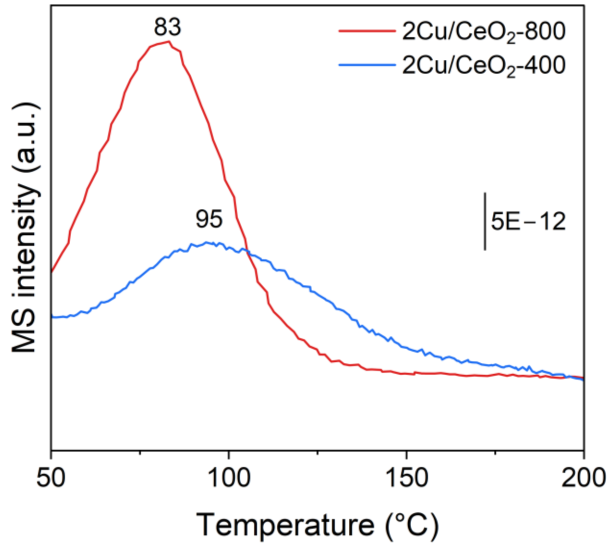

Fig. S21. C_2H_2-TPD characterization. The C_2H_2-TPD profiles of 2Cu/CeO_2-400 and 2Cu/CeO_2-800.

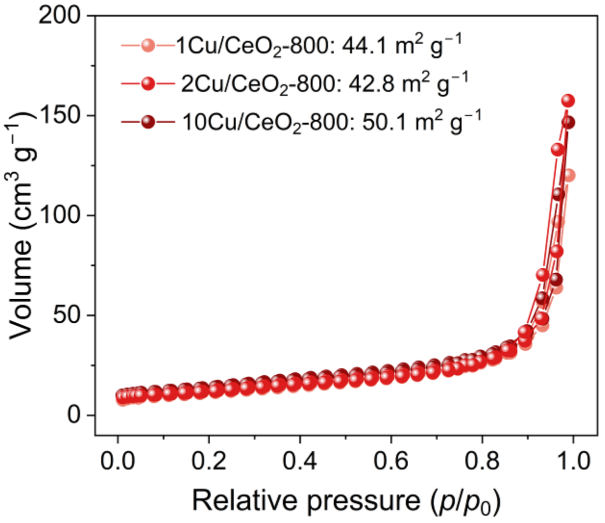

Fig. S22. Surface area characterization. N_2 sorption isotherms of 1Cu/CeO_2-800, 2Cu/CeO_2-800, and 10Cu/CeO_2-800. The surface area was calculated via the BET method.

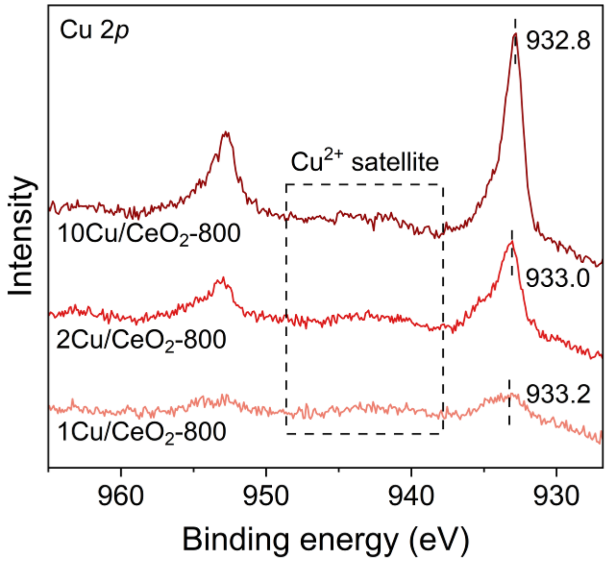

Fig. S23. XPS characterization. XPS spectra of 1Cu/CeO_2-800, 2Cu/CeO_2-800, and 10Cu/CeO_2-800.

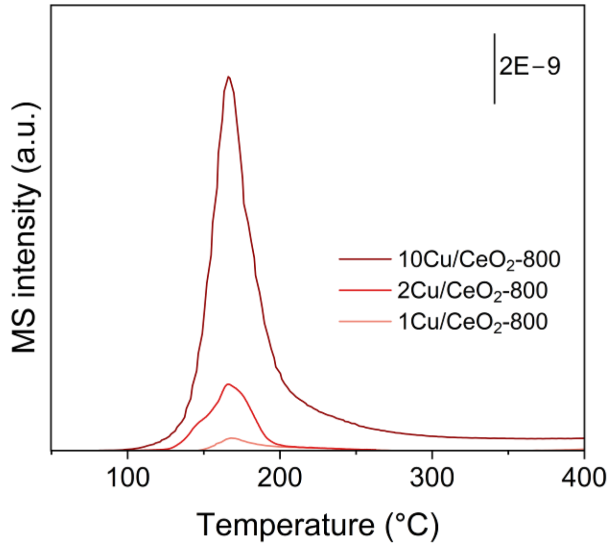

Fig. S24. H_2-TPR characterization. The H_2-TPR profiles of 1Cu/CeO_2-800, 2Cu/CeO_2-800, and 10Cu/CeO_2-800.

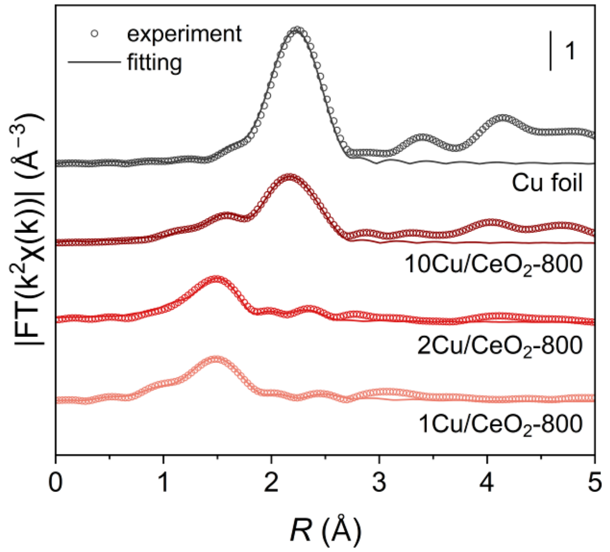

Fig. S25. EXAFS characterization. EXAFS data of Cu foil, reduced 1Cu/CeO_2-800, reduced 2Cu/CeO_2-800, and reduced 10Cu/CeO_2-800.

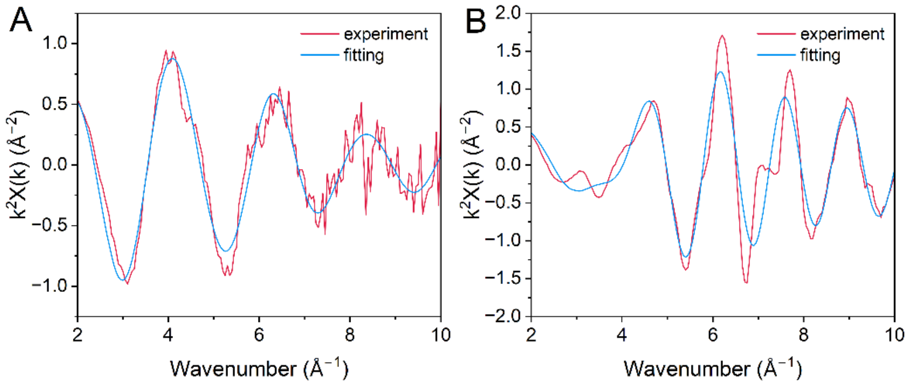

Fig. S26. Additional information on EXAFS. (**A** and **B**) The *k*-range profiles of EXAFS on reduced 1Cu/CeO_2-800 (A) and reduced 10Cu/CeO_2-800 (B).

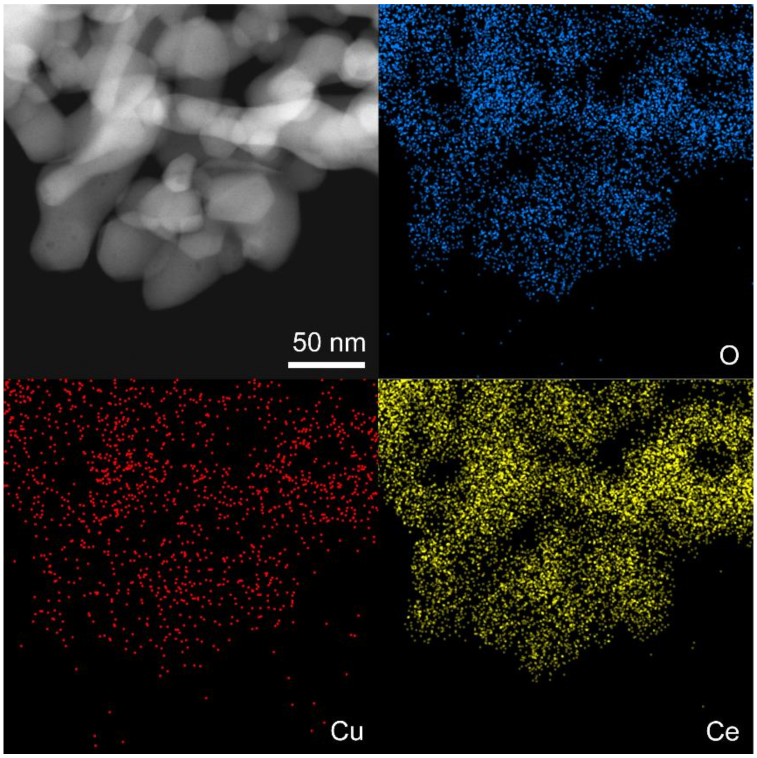

Fig. S27. HRTEM imaging and elemental analysis. HRTEM image and EDS-mapping of 2Cu/CeO_2-800 after stability test.

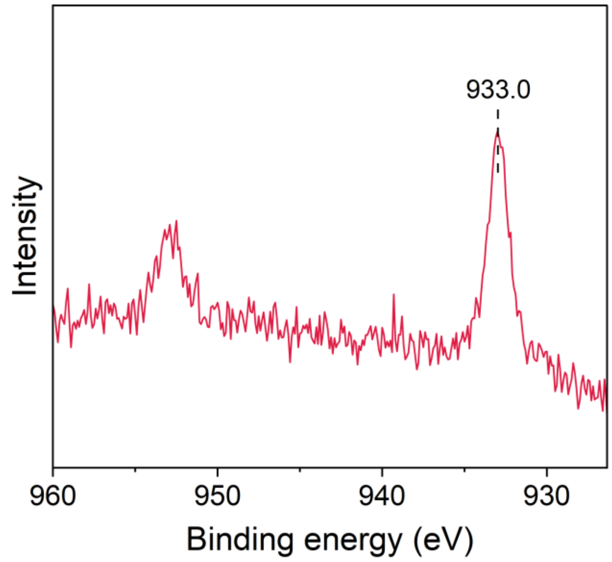

Fig. S28. XPS characterization. The *quasi-in-situ* XPS spectrum of 2Cu/CeO_2-800 after stability test.

Table S1. Fitting parameters of coordination numbers (CN), distances (*R*), energy shifts (ΔE_0), disorder factors (σ^2), and R factors for the EXFAS data.

Sample	Path	CN	*R* (Å)	ΔE_0 (eV)	σ^2	R factor
reduced 1Cu/CeO_2-800	Cu–O	3.5 ± 0.2	1.94 ± 0.0	−2.0	0.005	0.014
	Cu–Cu	0.3 ± 0.2	2.55 ± 0.01	5.0	0.010	
reduced 2Cu/CeO_2-800	Cu–O	2.6 ± 0.1	1.94 ± 0.01	−1.5	0.003	0.017
	Cu–Cu	1.0 ± 0.2	2.53 ± 0.02	−5.0	0.007	
reduced 10Cu/CeO_2-800	Cu–O	1.5 ± 0.2	1.96 ± 0.01	3.0	0.005	0.009
	Cu–Cu	6.2 ± 0.3	2.53 ± 0.00	−3.0	0.008	
fresh 2Cu/CeO_2-800 (25 °C, air)	Cu–O	3.4 ± 0.2	1.96 ± 0.01	2.8	0.002	0.020
	Cu–O	3.1 ± 0.6	2.80 ± 0.02	2.8	0.002	
2Cu/CeO_2-800 (200 °C, H_2, 0–15 min)	Cu–O	3.3 ± 0.2	1.96 ± 0.01	3.0	0.002	0.013
	Cu–O	3.0 ± 0.4	2.88 ± 0.01	3.0	0.002	
2Cu/CeO_2-800 (200 °C, H_2, 15–30 min)	Cu–O	1.0 ± 0.1	1.87 ± 0.01	3.0	0.001	0.009
	Cu–Cu	6.1 ± 0.3	2.49 ± 0.00	3.0	0.012	
	Cu–Cu	0.6 ± 0.1	2.93 ± 0.01	3.0	0.001	

$2Cu/CeO_2$-800 (200 °C, H_2, 30–45 min)	Cu–O	0.3 ± 0.2	2.04 ± 0.06	3.0	0.005	0.016
	Cu–Cu	12.0 ± 0.5	2.49 ± 0.00	3.0	0.015	
$2Cu/CeO_2$-800 (200 °C, H_2, 45–60 min)	Cu–Cu	11.1 ± 0.2	2.54 ± 0.00	4.5	0.010	0.003
$2Cu/CeO_2$-800 (200 °C, H_2, 60–75 min)	Cu–Cu	11.8 ± 0.3	2.54 ± 0.00	4.0	0.010	0.007
$2Cu/CeO_2$-800 (200 °C, H_2, 75–90 min)	Cu–Cu	11.9 ± 0.3	2.54 ± 0.00	4.0	0.010	0.008
